# Advancements in Water‐Saving Strategies and Crop Adaptation to Drought: A Comprehensive Review

**DOI:** 10.1111/ppl.70332

**Published:** 2025-07-02

**Authors:** Juan D. Franco‐Navarro, Yaiza Gara Padilla, Sara Álvarez, Ángeles Calatayud, José Manuel Colmenero‐Flores, María José Gómez‐Bellot, José Antonio Hernández, Isabel Martínez‐Alcalá, Consuelo Penella, Juan Gabriel Pérez‐Pérez, María Jesús Sánchez‐Blanco, María Tasa, José Ramón Acosta‐Motos

**Affiliations:** ^1^ Plant Ion and Water Regulation Group Instituto de Recursos Naturales y Agrobiología de Sevilla (IRNAS, CSIC) Seville Spain; ^2^ Hygiene Quality and R&D Department CLECE S.A., University Hospital of Puerto Real (HUPR) Cádiz Spain; ^3^ Departament de Biologia, Bioquimica I Ciències Naturals Universitat Jaume I, Avinguda de Vicent Sos Baynat Castelló Spain; ^4^ Unit of Woody and Horticultural Crops Instituto Tecnológico Agrario de Castilla y León (ITACyL) Valladolid Spain; ^5^ Departamento de Horticultura Instituto Valenciano de Investigaciones Agrarias, Centro de Citricultura y Producción Vegetal Moncada Spain; ^6^ Irrigation Department Centro de Edafología y Biología Aplicada del Segura (CEBAS‐CSIC) Murcia Spain; ^7^ Group of Fruit Trees Biotechnology, Department of Plant Breeding CEBAS‐CSIC Murcia Spain; ^8^ Plant Biotechnology, Agriculture and Climate Resilience Group, UCAM‐CEBAS‐CSIC Associated Unit to CSIC by CEBAS‐CSIC Murcia Spain; ^9^ Social Responsibility, Sustainability and Innovation Research Group Universidad Católica de Murcia Murcia Spain; ^10^ Centre for the Development of the Sustainable Agriculture Valencian Institute for Agricultural Research (CDAS‐IVIA) Moncada Spain; ^11^ Irrigation Advisory Service Institute for Agricultural Research (STR‐IVIA) Moncada Spain; ^12^ Plant Biotechnology for Food and Agriculture Group (BioVegA^2^) Universidad Católica San Antonio de Murcia (UCAM) Murcia Spain

**Keywords:** agriculture technologies, climate change, crop production, drought stress, global warming, modern farming, plant breeding, plant resilience, soil management, sustainable agriculture

## Abstract

Drought stress, which is one of the most critical environmental constraints affecting global crop productivity, is exacerbated by climate change and increased atmospheric water demand. This review comprehensively examines plant responses to drought, integrating physiological, morphological, biochemical, and genetic adaptations that contribute to water‐use efficiency and stress tolerance. Key mechanisms such as osmotic adjustment, stomatal regulation, antioxidant defense, and hormonal signaling are analyzed, highlighting their role in mitigating drought‐induced cellular damage. Advances in plant breeding and biotechnological approaches, including transgenic strategies, genome editing, and marker‐assisted selection, are discussed in the context of improving drought resilience. The importance of root system architecture, leaf anatomical modifications, and stress‐responsive transcription factors is underscored as essential components of drought adaptation. Additionally, agronomic innovations such as precision irrigation, soil management techniques, and plant‐microbe interactions are reviewed due to their potential to enhance sustainable water use in agriculture. The role of epigenetic modifications and long‐distance signaling mechanisms in drought acclimation is explored, shedding light on emerging strategies for engineering multi‐stress tolerant crops. Furthermore, we assess the impact of drought on crop nutritional quality, the trade‐offs between drought tolerance and pest resistance, and the socio‐economic implications of water scarcity on global food security. This review provides a roadmap for integrating cutting‐edge scientific knowledge with practical agricultural applications, aiming to develop resilient cropping systems capable of sustaining productivity under increasingly unpredictable climatic conditions.

## 1 Introduction

Drought stress is one of the most critical abiotic factors limiting global crop productivity and threatening food security. The increasing frequency and severity of drought events, exacerbated by climate change, have intensified the need for developing resilient crops capable of sustaining growth under water‐limited conditions (Acosta‐Motos et al. 2024). Understanding how plants respond and adapt to drought is essential for designing effective mitigation strategies that integrate physiological, genetic, and agronomic approaches. Drought stress refers to a prolonged deficiency of water in a specific area, impacting natural ecosystems, agriculture, and human socio‐economic systems (dos Santos et al. 2022; Wu et al. 2022). At its core, drought stress manifests as a shortage of water relative to normal conditions for a region over a specific period of time (Cook et al. 2018; Vicente‐Serrano et al. 2020). This scarcity of moisture can be a gradual process, occurring over months or years in the form of meteorological droughts, or it can occur suddenly with immediate and severe impacts, a phenomenon known as a flash drought. The duration and intensity of droughts can vary widely, ranging from mild, localized water shortages to extreme droughts covering vast geographical regions, sometimes even entire countries (Christian et al. 2023; Rahim et al. 2023; Rakkasagi et al. 2023; Yuan et al. 2023).

The global prevalence of drought stress is not uniform. It tends to affect arid and semi‐arid regions more severely due to their already low precipitation levels. However, no region is entirely immune to the effects of drought, especially in the context of climate change, which has exacerbated the frequency and intensity of drought events. The Intergovernmental Panel on Climate Change (IPCC) predicts that, due to global warming, many regions will face increasingly severe droughts in the coming decades (Caretta et al. 2022; Li, Wang, et al. 2023; Li, Piao, et al. 2023; Mani et al. 2024). Climate change, driven by the accumulation of greenhouse gases such as carbon dioxide (CO_2_), methane, and nitrous oxide (N_2_O) in the atmosphere, is fundamentally altering weather patterns and disrupting the Earth's hydrological cycle. Rising global temperatures due to climate change are leading to shifts in atmospheric circulation patterns, which in turn affect the distribution and intensity of precipitation (Douville et al. 2021; Etukudoh et al. 2024; Medellín‐Azuara et al. 2024). This dual impact of less frequent but more intense rainfalls, combined with higher evaporation, reduces soil moisture and impairs plant growth, further exacerbating the drought stress experienced by ecosystems and agricultural systems. In arid and semi‐arid regions like the Mediterranean basin, parts of the United States, Sub‐Saharan Africa, and Australia are predicted to experience more frequent and intense droughts due to climate change. These regions are crucial to global agriculture, and any disruption to their water resources can have ripple effects on global food security (Trenberth et al. 2014; Vicente‐Serrano et al. 2020).

Agriculture is perhaps the sector most vulnerable to the impacts of drought stress. Crops require water at various stages of growth to maintain their physiological processes, and water scarcity can severely impede these processes. Under drought conditions, reduced soil moisture levels limit water uptake by plant roots, leading to impaired nutrient absorption, stunted growth, reduced yields, and ultimately, decreased food production (Álvarez and Acosta‐Motos 2022; Orimoloye 2022). Some of the most drought‐sensitive crops include cereals such as maize and wheat, as well as horticultural crops such as fruits and vegetables, which are critical for global food security and nutrition (Daryanto et al. 2016; Liaqat et al. 2024). The effects of drought stress are not limited to crop yields alone. Drought also impacts the quality of agricultural products. For instance, drought‐stressed plants may produce smaller, less nutritious fruits and vegetables (Khatun et al. 2021; Zandalinas et al. 2018). Additionally, crops grown under drought conditions may be more vulnerable to pests and diseases, further compounding the negative impacts on agricultural output (Kaushik et al. 2023; Moghaddam et al. 2024). However, this relationship between drought and loss of nutritional quality of fruits and vegetables does not always occur. Higher fruit quality was reported under moderate drought conditions in, for example, citrus (Khan, Awan, et al. 2023; Khan, Liu, et al. 2023; Khan, Liu, et al. 2023), tomato (Gao et al. 2023), and peaches and grapes (Liu et al. 2023). In the same way, it is observed that drought tolerance sometimes has a negative impact on pest resistance, making wheat plants, for example, more susceptible to aphids (Ramírez et al. 2023). Inversely, poplar trees (*Populus* sp.) subjected to water scarcity are more resistant against bacteria and fungi pathogens, and their spread through the tree is less likely due to cavitation phenomena in their xylem vessels (Rosso et al. 2023).

Plants have evolved a range of mechanisms to cope with drought, including stomatal regulation to control water loss, osmotic adjustments to maintain cellular hydration, and antioxidant defenses to counteract oxidative stress (Arve et al. 2011; Farooq et al. 2009; Franco‐Navarro et al. 2016). Additionally, modifications in root architecture and hormonal signaling play key roles in enhancing water uptake and stress tolerance (Kalra et al. 2024; Shoaib et al. 2022; Zhou et al. 2024). These physiological responses are regulated at the molecular level through stress‐induced gene expression, epigenetic modifications, and complex signaling pathways that coordinate adaptive mechanisms. Advancements in plant breeding and biotechnology have facilitated the identification and manipulation of genes associated with drought tolerance (Parmar et al. 2017; Sakuma et al. 2002; Umezawa et al. 2006). Marker‐assisted selection, genome‐wide association studies, and CRISPR‐based genome editing have enabled the development of drought‐resistant crop varieties with improved water‐use efficiency (Patel and Mishra 2021; Pellegrineschi et al. 2004). Additionally, epigenetic regulation and transgenic approaches offer promising strategies to enhance stress memory and improve long‐term resilience in agricultural systems. Beyond genetic improvements, agronomic innovations play a crucial role in mitigating drought stress. Precision irrigation techniques, soil moisture conservation practices, and beneficial plant‐microbe interactions are being increasingly recognized for their potential to optimize water availability and improve crop performance under drought conditions (Ruiz‐Lozano et al. 2012; Shankar and Moorthi 2024; Vallejo‐Gómez et al. 2023). Integrating these strategies with advanced breeding technologies is essential for developing sustainable solutions to drought‐related challenges.

In conclusion, drought stress, intensified by climate change, represents a critical global challenge with profound implications for agriculture and ecosystems. Addressing this issue requires a comprehensive, multi‐faceted approach that integrates both adaptation and mitigation strategies (Acosta‐Motos et al. 2024; Grigorieva et al. 2023; Vadez, Messina, et al. 2023). By leveraging genetic advancements, biotechnological and modern farming technological innovations, and sustainable water management techniques, we can significantly improve crop resilience to drought and safeguard food security for future generations. This review aims to thoroughly explore these strategies, offering a detailed understanding of current advancements and potential opportunities for addressing drought stress in crops, while providing insights into sustainable agriculture.

## 2 Chapter 1—Physiological and Morphological Adaptations to Cope With Drought

**FIGURE 1:**
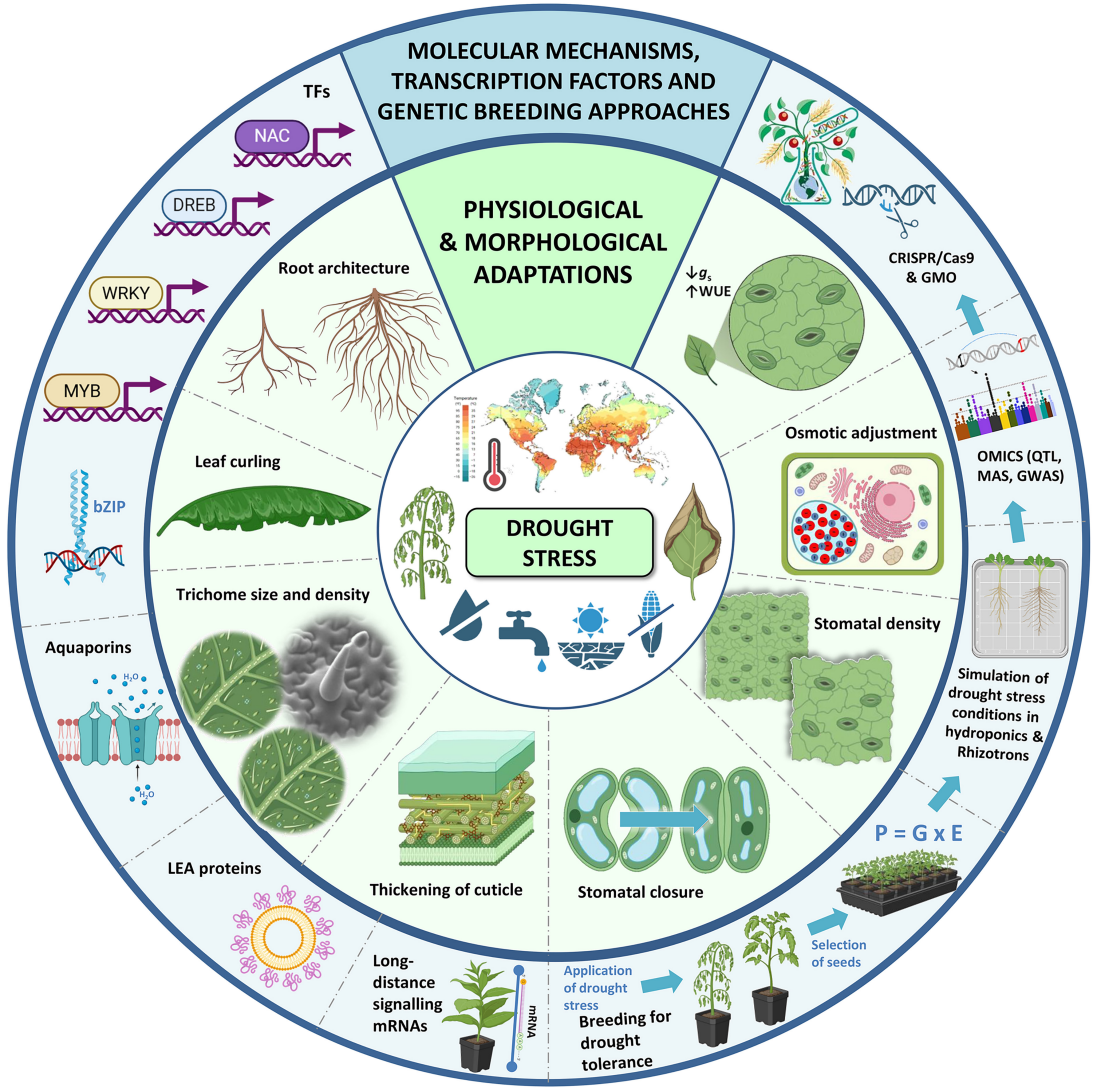
Schematic representation of the main aspects of Chapter 1 (Physiological and morphological adaptations to cope with drought) and Chapter 2 (Mechanisms and approaches to enhance drought tolerance: Molecular, transcriptional, and genetic perspectives). E, Environment; GMO, genetically modified organisms; G, genotype; P, phenotype; *g*_s_, stomatal conductance; TFs, transcription factors; WUE, water‐use efficiency. Parts of the images were fully provided with permission from J.D. Franco‐Navarro's thesis (Franco‐Navarro 2022). *Sources:* Most elements of this scheme were created with BioRender.com (CC‐BY 4.0 license).

Physiological and morphological adaptations are crucial for enhancing crop survival and productivity under drought conditions (Kapoor et al. 2020). These adaptations include changes in water use efficiency (WUE), stomatal regulation, and osmotic adjustment, which help plants maintain water balance during periods of drought. Additionally, morphological traits such as deeper root systems, thicker cuticles, and smaller leaves reduce water loss and improve access to soil moisture (Figure 1).

In this context, plants employ three primary resilience mechanisms to cope with drought: drought tolerance, drought resistance, and cellular dehydration avoidance. Drought tolerance refers to a plant's ability to maintain it's physiological functions despite water deficits (WD), often through osmotic adjustments, antioxidant production, and metabolic flexibility (Bechtold 2018; Boyer 1996; Shinozaki and Yamaguchi‐Shinozaki 2007; Umezawa et al. 2006). Drought resistance encompasses strategies that limit water loss and enhance water uptake, such as stomatal regulation, deep root systems, and cuticle reinforcement (Fang and Xiong 2015; Hu et al. 2006; Hu and Xiong 2014; Nieves‐Cordones et al. 2019; Xiao et al. 2007). Cellular dehydration avoidance involves mechanisms that prevent cells from reaching critical dehydration levels, including increased leaf succulence, accumulation of compatible solutes, and structural modifications that reduce transpiration (Gowda et al. 2011; Kooyers 2015; Mahouachi et al. 2014; Souza Santana‐Vieira et al. 2016). These adaptive responses enable plants to survive and persist under prolonged drought conditions, although they often come with trade‐offs in growth and productivity.

Understanding and harnessing these natural plant responses is essential for developing drought‐tolerant crops, as they provide a foundation for breeding and genetic modification efforts aimed at improving agricultural resilience in arid environments (Kapoor et al. 2020; Rosero et al. 2020).

### 2.1 Root Architecture

The development of deeper root systems in crops under drought conditions plays a crucial role in enhancing their drought resistance, as they enable plants to access water stored in deeper soil layers. This adaptation mechanism allows plants to explore a larger soil volume and extract water from deeper reservoirs, significantly contributing to their resilience to water scarcity. Some studies have highlighted the importance of root architecture in drought avoidance and the potential for introducing or manipulating specific genes, such as *DRO1*, to enhance drought resistance in crops (Kalra et al. 2024). Efforts to understand the genetic, epigenetic, and metabolic bases of traits associated with drought resistance in crops are essential for advancing crop breeding for improved drought resilience and ensuring global food security (Raza et al. 2023).

Root morphology also plays a critical role in enhancing water absorption from the soil under drought conditions. Crops exhibit changes in root morphology, such as increased root length, density, and surface area, to maximize water uptake efficiency. Fine root hairs and lateral roots are particularly important for enhancing water uptake efficiency (Zhou et al. 2024). Research has shown that narrow root angles result in downward root growth, leading to deep rooting and better yield under drought conditions in crops such as cotton (Guo et al. 2024). The development of deep root systems, an increased root length density in medium and deep soil layers, a reduced root length density in the topsoil, an increased root hair growth, and an increased xylem diameter are proposed as an ideal drought‐resistant ideotype (Kalra et al. 2024). The “steep, cheap, and deep” ideotype emphasizes efficient moisture uptake from the subsoil, while the “wide, shallow, and fine” ideotype focuses on capturing water from low rainfall events before it is lost by evaporation. Understanding the effect of deep roots on biological tillage, creation of biopores, and carbon input in soil, and their impacts on soil water storage, subsequent crops, and long‐term drought resilience, is crucial for developing drought‐tolerant crops (Shoaib et al. 2022). Sustaining root growth in dry soil requires efficient root osmoregulation under WD, a process regulated by abscisic acid (ABA). This phytohormone represses the expression of the SKOR channel, reducing K^+^ release into the xylem (Gaymard et al. 1998). ABA also represses *AtSLAH1* expression, shifting SLAH3/SLAH1 heteromers to SLAH3/SLAH3 homomers, which favour N$O_{3}^{-}$ over Cl^−^ transport (Cubero‐Font et al. 2016). These mechanisms retain K^+^ and Cl^−^ in the root, which are critical for supporting root growth under WD.

### 2.2 Leaf Morphology and Anatomy

#### 2.2.1 Leaf Curling

To reduce water loss through transpiration, leaves may curl or roll, reducing the leaf surface area exposed to the atmosphere. This is a common phenomenon described in the fight against drought stress in higher plants such as Quercus (Abrams 1990), rice (Latif et al. 2023; Opalofia et al. 2018), sugarcane (Zhang, Zhang, Du, et al. 2015; Zhang, Zhang, Xia, et al. 2015), and soybean (Chun et al. 2021). Curiously, this morphological modification of the leaves can be promoted by a virus infection, promoting tolerance to drought, as it was observed in tomato plants infected with the Tomato yellow leaf curl virus (TYLCV; Shteinberg et al. 2021).

#### 2.2.2 Changes in Trichome Density and Size

Some plants develop trichomes, specialized epidermal unicellular or multicellular glandular and non‐glandular appendages (Bickford 2016). Their main role is to defend against herbivores and pathogens through physical means or secreted chemical compounds (Allen et al. 1991; Hanley et al. 2007; Stavrianakou et al. 2010; Wagner 1991). Another important role is related to the capacity of regulating water relations on leaf surfaces and the mesophyll. In this way, trichomes can reduce transpiration rates by creating a boundary layer of still air around the leaf surface, thereby reducing water loss (Bickford 2016; Schreuder et al. 2001). In addition, trichomes play an important role in reducing DNA mutations and PSII photochemistry imbalance due to UV‐B damage, as observed in quercus and *Artotheca* leaves (Karabourniotis and Bornman 1999; Ripley et al. 1999; Skaltsa et al. 1994), or in *Olea* leaves (Grammatikopoulos et al. 1994). Changes in temperature, humidity, vapor pressure deficit (VPD), nutrient uptake, and water availability increase leaf trichome density (Ehleringer 1982; Franco‐Navarro et al. 2016; Nazari et al. 2018), mainly in the abaxial surfaces of the leaf (Rotondi et al. 2003; Schreuder et al. 2001). A pubescent and dense trichome layer in xerophytic plants is an adaptation against drought tolerance, high temperatures, and water scarcity (Bickford 2016; Ehleringer 1982), and environmental conditions that are linked to desert and Mediterranean areas (Bickford 2016; Ehleringer 1982).

#### 2.2.3 Cuticle Thickening

The cuticle, a waxy layer on leaves typically composed of n‐alkanes, fatty acids, aldehydes, primary alcohols, secondary alcohols, ketones, and esters (Jenks 2002), which covers the leaf surface, is a plant adaptation that enhances plant survival in severe environments. Changes in the climate surrounding the plant through, that is, drought stress (Goodwin and Jenks 2005; Guzmán‐Delgado et al. 2021), or water scarcity and high temperatures in arid environments (Schuster et al. 2016), promote changes in the composition of the plant cuticle. Prolonged exposure to drought conditions induces a wide expression of genes involved in cuticle synthesis that may thicken this layer to reduce water loss through transpiration, thus minimizing dehydration under drought conditions (Goodwin and Jenks 2005).

### 2.3 Stomatal Regulation

Stomata are small leaf pores flanked by two specialized epidermal cells called guard cells (Lawson and Leakey 2024; Taiz and Zeiger 2002; Supplementary Figure S1A,B). Stomata are found in the abaxial and adaxial epidermis of leaves, in some young stems of higher plants, and in some organs of mosses and liverworts. Their functions in plants are: gas exchange (CO_2_ uptake, water (H_2_O) vapour efflux), maintaining an adequate water balance, nutrient uptake, and photosynthesis (Farooq et al. 2009). This is achieved by this plant organ through the activity of the transporters and ion channels present in the guard cells, which regulate the turgor of these cells and promote the opening and closing of the stomata (Roux and Leonhardt 2018). For more information, the work by Saito and Uozumi (2019) and previous bibliography (Hedrich and Geiger 2017; Konrad et al. 2018) are recommended.

#### 2.3.1 Stomatal Opening/Closure Behavior

Stomatal opening requires the active transport of proton (H^+^) ATPases, which hyperpolarize the plasma membrane (PM) to activate the passive entry of K^+^ through rectifier channels and the active entry of chloride (Cl^−^) or nitrate (N$O_{3}^{-}$), coupled to the movement of H^+^ through transporters of the NPF (Nitrate and peptide transporter family; Guo et al. 2003; Wen et al. 2017). The accumulation of inorganic ions and organic molecules, such as malate (Mal^2−^) or fumarate (Lee et al. 2008), leads to the movement and accumulation of vacuolar water that leads to an increase in cell volume, causing the change in conformation of the guard cells, opening the pore of the stoma. An H^+^ gradient in the vacuole is required for this accumulation of inorganic ions, which is achieved with the H^+^ V‐ATPase pump of the tonoplast. This induces the activation of specific Cl^−^ (and/or N$O_{3}^{-}$) transporters and channels from the ALMT (Aluminium activated malate transporter), DTX/MATE (Detoxification efflux carrier or multidrug and toxic compound extrusion protein), and CLC (Cation‐chloride cotransporters) families (Saito and Uozumi 2019; Figure S1C).

Stomatal closure requires a reversal of the previously described ion fluxes, that is, the efflux of Cl^−^, N$O_{3}^{-},$ and Mal^2−^, which depolarizes the PM and causes the efflux of K^+^ through voltage‐rectifying channels, with the consequent loss of water and turgor in the guard cells surrounding the stomatal opening, leading to stomatal closure (Saito and Uozumi 2019). Stomatal closure is initiated by the activation of channels from the SLAC/SLAH (Slow‐type [S‐type] anion channels/SLAC1 homologues), ALMT, and CLC families, in response to the lack of light, the circadian rhythm, and the perception of ABA in conditions of WD (Jossier et al. 2010; Ramesh et al. 2015; Saito and Uozumi 2019; Zhang et al. 2001). Several secondary messengers in the stomatal closure pathway also contribute to this, such as calcium ions (Ca, Ca^2+^; Atkinson et al. 1990), hydrogen peroxide (H_2_O_2_; Zhang et al. 2001) and nitric oxide (NO; García‐Mata and Lamattina 2001; Neill et al. 2002; Figure S1C).

Under drought stress, plants force the closure of stomata only when the benefits like water retention, reduction in evapotranspiration, and water saving outweigh the following negative effects: reduction in water content, nutrients and CO_2_ uptake, reduction in photosynthesis, transpirational cooling, and growing. Stomatal closure occurs as a consequence of an imbalance in the content of various phytohormones like ABA, jasmonic acid (JA), salicylic acid (SA), etc. It is also provoked by an excess of reactive oxygen species (ROS) such as H_2_O_2_, superoxide radicals (O^2·−^), and hydroxyl radicals (·OH^−^). Lastly, secondary messengers (NO, Ca^2+^, etc.) promoted by an induction of specific genes and transcription factors (TFs) decrease drought tolerance via stomatal closure (Ahammed et al. 2021; Liu et al. 2022; Pirasteh‐Anosheh et al. 2016; Shen et al. 2021; Xing et al. 2020).

#### 2.3.2 Stomatal Density

The efficiency of gas exchange through stomatal openings is determined by the size of the opening and also by the number of stomata per area, which is called the stomatal density (Metwally et al. 1971). The more stomata per area, the more exchanges of CO_2_ and H_2_O occur. Some species may alter stomatal density or size in response to drought, soil moisture, air humidity, nutrients (e.g., K^+^, Cl^−^, etc.), ABA content, and so on, regulating gas exchange and water loss without impairing photosynthetic efficiency (Farooq et al. 2009; Gray and Dunn 2024; Pirasteh‐Anosheh et al. 2016). Reducing stomatal density enhances drought tolerance, and this behavior has been observed in Arabidopsis (Xie et al. 2012), barley (Hughes et al. 2017), tobacco (Franco‐Navarro et al. 2021), poplar (Jiao et al. 2022), and rice (Caine et al. 2019), among other species.

### 2.4 Osmotic Adjustments to Overcome Drought

Osmotic adjustments are a critical adaptive mechanism that plants use to sustain cellular turgor pressure (Ψ_P_) and water balance during drought or water‐deficit conditions (Farooq et al. 2009). This process involves the accumulation of both organic and inorganic solutes, which help reduce the osmotic potential (Ψ_π_) of the cytoplasm, allowing plants to continue absorbing water from the soil and avoid dehydration. Compatible organic solutes (Patakas et al. 2002), or osmolytes, include compounds such as sugars sucrose or trehalose; Kameli and Lösel 1995), amino acids like proline and glycine betaine; Meloni et al. 2001), and polyols, which act to stabilize proteins and membranes under stress (Farooq et al. 2009). Inorganic solutes, such as Cl^−^, N$O_{3}^{-}$, and K^+^, are also sequestered in the vacuoles to contribute to the osmotic balance (Chen and Jiang 2010; Patakas et al. 2002).

By lowering the Ψ_π_, these solutes enable plants to maintain a favorable gradient for water movement, supporting cellular hydration and turgor, which is essential for sustaining growth and metabolic functions during periods of drought. Osmotic adjustments also play a role in protecting cell membranes, stabilizing protein structures, and preventing oxidative damage (Chen and Jiang 2010; Farooq et al. 2009). Furthermore, these adjustments contribute toward the maintenance of stomatal function, enabling plants to regulate gas exchange and photosynthesis, which are often impaired under drought conditions (Flowers et al. 2015).

### 2.5 Abscisic Acid (ABA)

ABA is a crucial phytohormone in plant responses to drought stress, increasing under water‐deficit conditions to trigger physiological and molecular adaptations (Kim et al. 2010; Taiz and Zeiger 2002). Synthesized in roots and leaves, ABA promotes stomatal closure by regulating ion fluxes in guard cells, minimizing water loss (Arve et al. 2011; Kang et al. 2010). It also enhances drought tolerance by encouraging deeper root growth while suppressing shoot growth to optimize water uptake (Rowe et al. 2016). Additionally, ABA modulates gene expression, activates ABA‐responsive TFs (AREBs/ABFs) that regulate genes encoding antioxidant enzymes such as superoxide dismutase (SOD) and catalase (CAT), which mitigate oxidative stress during drought (Hussain et al. 2021; Liao et al. 2019). It also upregulates protective proteins like late embryogenesis abundant (LEA) and heat shock proteins (HSPs), stabilizing cellular structures (Garay‐Arroyo et al. 2000; Hernández‐Sánchez et al. 2022; Reyes et al. 2005). Furthermore, ABA influences osmoprotectant synthesis, including proline and sugars, which maintain cellular water balance (Kim et al. 2024). Through interactions with other hormones like ethylene, JA, and SA, ABA fine‐tunes stress responses, ensuring coordinated drought adaptation (Rowe et al. 2016; Singh and Roychoudhury 2023). Thus, ABA serves as a master regulator of drought tolerance by integrating immediate physiological responses with long‐term genetic regulation (Fujita et al. 2005; Qiao et al. 2024; Wang et al. 2020).

ABA transport into guard cells occurs via plasma membrane ABC transporters, where it binds to receptors such as PYR/PYL/RCAR and GCR2, initiating a signaling cascade that activates ion channels, leading to ion efflux (K^+^, Cl^−^, N$O_{3}^{-}$, and Mal^2−^) and stomatal closure (Castillo et al. 2015; Klingler et al. 2010; Liu et al. 2022; Rodriguez et al. 2019). Disruptions in these receptors impair stomatal closure under drought, highlighting their essential role (Kang et al. 2010). ABA also induces ROS and NO production, which modulate H^+^‐ATPase and Ca^2+^ pumps, increasing cytosolic Ca^2+^. This activates anion channels, leading to K^+^ efflux, maintaining membrane depolarization, and promoting stomatal closure (Wasilewska et al. 2008). Mal^2−^, a key osmolyte, converts to starch, lowering Ψ_π_, reducing Ψ_P_, and ensuring water conservation (Kim et al. 2010). These biochemical events underscore ABA's complex role in regulating stomatal function under drought conditions.

In response to WD, plants undergo osmotic adjustments by rapidly accumulating inorganic ions like K^+^ and Cl^−^ in vacuoles, maintaining Ψ_π_ and Ψ_P_ (Shabala et al. 2000; Shabala and Lew 2002). These ions act as efficient osmoregulatory molecules due to their availability and mobility (Colmenero‐Flores et al. 2019). ABA also promotes the accumulation of osmolytes such as proline, glycine betaine, sugars (e.g., sucrose and trehalose), and polyols, which lower Ψ_π_, stabilize proteins and membranes, and protect against dehydration (Cardoso et al. 2020; Sharma et al. 2019). ABA upregulates genes involved in osmolyte biosynthesis, including proline synthesis enzymes like P5CS and sugar metabolism enzymes such as TPS1 for trehalose biosynthesis (Nanjo et al. 1999; Romero et al. 1997; Serrano et al. 1999; Yang et al. 2015). Glycine betaine accumulation protects the photosynthetic machinery and scavenges ROS, further enhancing drought tolerance (Parmar et al. 2017; Xian et al. 2014). Additionally, ABA regulates ion transporters and aquaporins to manage water flow across membranes, ensuring cellular hydration (Maurel et al. 2021). Through these mechanisms, ABA plays a pivotal role in plant survival during prolonged drought conditions.

### 2.6 Transpiration Efficiency

Transpiration efficiency refers to the ratio of carbon assimilation (photosynthesis) to water loss through transpiration, representing the plant's ability to maximize biomass production per unit of water consumed. This efficiency is crucial for plant survival and productivity, especially in environments with limited water resources. Under drought stress, plants may exhibit significant alterations in transpiration efficiency to optimize water use, often by minimizing water loss while maintaining adequate carbon assimilation rates (Hatfield and Dold 2019; Petrík et al. 2023).

Plants can enhance transpiration efficiency through various physiological and morphological adjustments. For instance, they may regulate stomatal conductance (*g*_s_) by partially closing stomata to reduce transpiration without entirely compromising photosynthesis. This balance allows plants to conserve water while still assimilating CO_2_ for growth. Additionally, modifications in leaf anatomy, such as a reduction in stomatal density or increased cuticle thickness, can further reduce water loss while maintaining photosynthetic capacity (Farooq et al. 2024).

Furthermore, some plants exhibit shifts in photosynthetic pathways under drought stress, such as moving from C_3_ to C_4_ or CAM (Crassulacean acid metabolism) photosynthesis, which are more water‐efficient (Wang, Zhou, et al. 2024). These adaptations allow for improved transpiration efficiency by capturing more carbon per unit of water used, thus enhancing biomass production under water‐limited conditions. An enhanced root architecture, which increases water uptake from deeper soil layers, also contributes to maintaining transpiration efficiency during prolonged drought periods (Karami et al. 2023; Tan and Chen 2023).

By optimizing transpiration efficiency, plants not only improve their WUE but also sustain growth and productivity in challenging environments, making this trait an essential component of drought tolerance.

### 2.7 Stomatal Conductance (*g*_s_)

Drought‐adapted plants regulate *g*_s_ to balance water loss with photosynthetic CO_2_ uptake, a critical adaptation for survival in water‐limited environments (Vadez et al. 2024; Wang, Zhou, et al. 2024). *g*_s_ refers to the rate at which CO_2_ enters and water vapour exits the leaf through stomata, and it plays a central role in determining the plant's WUE. By adjusting the aperture of stomata, plants can minimize water loss through transpiration while maintaining an adequate supply of CO_2_ for photosynthesis (Lawson and Blatt 2014; Petrík et al. 2023).

Under drought conditions, plants typically reduce *g*_s_ by partially or fully closing their stomata. This helps limit water loss, particularly during peak daylight hours when transpiration rates are highest. However, closing stomata also restricts CO_2_ uptake, which can negatively affect photosynthetic rates. To mitigate this, drought‐adapted plants often employ strategies that allow them to maintain carbon fixation with reduced stomatal opening (Wang, Zhou, et al. 2024). For example, some plants may increase the concentration of CO_2_ in the mesophyll cells, enhancing the efficiency of carbon assimilation even with a lower *g*_s_ (Guadarrama‐Escobar et al. 2024).

Plants also regulate *g*_s_ through hormonal signals, primarily ABA, which is rapidly synthesized in response to WD. ABA triggers the closing of stomata by inducing ion fluxes in guard cells, leading to a reduction in their Ψ_P_. This physiological response helps plants conserve water during drought events, but is finely tuned to avoid excessive closure that could severely limit photosynthesis (Manandhar et al. 2024). Additionally, long‐term drought stress can lead to structural changes in stomatal characteristics, such as a reduction in stomatal density or changes in stomatal size, which further enhance water conservation. The reduction in stomatal density, and therefore *g*_s_, may be a consequence of an increase in epidermal cell volume due to osmotic processes under nutrition with Cl^−^ at macronutrient levels (Franco‐Navarro et al. 2016, 2021). Some species may also develop thicker cuticles and reduce their leaf area to decrease overall water loss while still maintaining essential gas exchange for photosynthesis (Gray and Dunn 2024).

In some cases, plants may shift their photosynthetic activity to different times of the day, such as early morning or late afternoon, when temperatures are lower and transpiration rates are reduced. This diurnal regulation of *g*_s_ enables plants to optimize water use and maintain photosynthetic efficiency despite limited water availability (Gao et al. 2005). Moreover, *g*_s_ is influenced by environmental factors such as humidity, light intensity, soil moisture levels, and plant nutrition (e.g., Cl^−^ nutrition), allowing plants to dynamically adjust their water‐use strategies in real time (Flexas et al. 2014; Franco‐Navarro et al. 2016; Lawson and Blatt 2014). By fine‐tuning stomatal behavior in response to fluctuating conditions, drought‐adapted plants can strike a balance between conserving water and maximizing carbon uptake, ensuring survival and productivity in challenging environments (Hommel et al. 2014; Osman et al. 2024).

### 2.8 Water‐Use Efficiency (WUE)

Water‐use efficiency (WUE) reflects the balance between carbon gain and water loss in plants, a concept introduced a century ago by Briggs and Shantz (1913); fully reviewed in Petrík et al. 2023). Since then, multiple methods have been developed to assess WUE at different levels of plant organization and time scales (Brendel 2021; Petrík et al. 2023; Vadez, Pilloni, et al. 2023), and due to the complexity of WUE traits, different concepts arise depending on the scale of measurement and the physiological processes involved (Table 1). Of these, WUEbio is considered the most accurate for evaluating plant resource use, as it accounts for both assimilatory and respiratory processes alongside productive and unproductive water losses (Brendel and Epron 2022). While WUEi is easier to measure, it only represents a single point in time and should not be the standard in agriculture, where balancing productivity and water resources is crucial (Condon et al. 2004; Flexas 2016; Flexas et al. 2016).

**TABLE 1:** Overview of different water use efficiency (WUE) concepts.

Concepts	Definition	References
WUEi	*A*_N_ *g*_s_^−1^ (μmol CO_2_ mol H_2_O^−1^)	Rosales et al. (2012)
Instantaneous WUE	*A*_N_ E^−1^ (μmol CO_2_ mmol H_2_O^−1^)	Bacon (2009)
WUEbio	B *ET* ^−1^ (kgDW m^3^ or gDW L^−1^)	Abbate et al. (2004), Brendel (2021), and Condon et al. (2004)
Yield WUE	Y *ET* ^−1^ (kg m^−3^ or ton m^−3^)	Hatfield and Dold (2019) and Zahoor et al. (2019)
WUE_growth_	BAI *T* ^−1^ (cm^2^ L^−1^)	Szatniewska et al. (2022)
WUE_13C_	BAI *T* ^−1^ (cm^2^ L^−1^)	Farquhar et al. (1989), Frank et al. (2015, Ma, Zhao, et al. (2023), and Ma, Yu, et al. (2023)
WUE_GPP_	GPP *ET* ^−1^ (g C kg H_2_O^−1^ d^−1^ or g C kg H_2_O^−1^ y^−1^)	Ahmadi et al. (2019) and Yi et al. (2019)

Abbreviations: Annual basal area increment, BAI; cm^2^ year^−1^; annual transpiration, *T*; L year^−1^ or mm year^−1^; crop, biomass or integrated WUE, WUE_bio_; carbon isotope ratio, 13C; δ^13^C; crop yield, Y; kg ha^−1^ or ton ha^−1^; gross primary production, GPP; g C m^−2^ d^−1^ or g C m^−2^ year^−1^; intrinsic or photosynthetic WUE, WUEi; leaf transpiration rate, E; mmol H_2_O m^−2^ s^−1^; ratio of CO_2_ assimilation or net photosynthetic rate, *A*_N_; μmol CO_2_ m^−2^ s^−1^; stomatal conductance, *g*_s_; mol H_2_O m^−2^ s^−1^; total dry biomass production, B; kgDW ha^−1^ or gDW m^−2^; total evapotranspiration, ET; mm or m^3^ ha^−1^.

Consequently, simpler and more specific WUE parameters are often required to identify viable targets for improvement (Flexas et al. 2016).

Given that agriculture consumes 80% of available freshwater, improving WUE is a priority for enhancing crop productivity while minimizing water loss. Efforts have focused on physiological and genetic factors affecting WUE (Blum 2009; Condon et al. 2004; Hessini et al. 2009; Medrano et al. 2015). Strategies to enhance WUE include optimizing irrigation, reducing soil evaporation, improving carbon fixation efficiency relative to transpiration, and directing more biomass to harvestable yield (Condon et al. 2004). Due to the complexity of these traits, simpler, more specific WUE parameters are often needed to identify viable targets for improvement (Flexas et al. 2016).

WUE is influenced by *g*_s_, leaf traits, and photosynthetic pathways. C_4_ and CAM plants enhance WUE by optimizing carbon fixation while minimizing water loss, unlike C_3_ plants, which lose more water through transpiration (Croce et al. 2024; Hah et al. 2022). Leaf morphology, including thickness and cuticle properties, also affects WUE, with a negative correlation observed between WUE and specific leaf area (SLA; Ferguson et al. 2024; Gago et al. 2014; Liu and Stützel 2004; Wright et al. 1994). Environmental factors such as soil moisture, temperature, and humidity further modulate WUE, with drought triggering physiological responses like osmolyte accumulation and root adjustments (Bhattacharya 2021). Nutrients, such as Cl^−^, can enhance WUE by reducing *g*_s_ without compromising carbon assimilation (*A*_N_), improving drought resistance (Franco‐Navarro et al. 2021; Franco‐Navarro et al. 2019; Franco‐Navarro et al. 2016).

For agriculture, high WUE improves yields with less irrigation, making crops better adapted to drought (Li et al. 2017; Yang et al. 2024), driving breeding and biotechnological efforts to enhance crop resilience amid climate change (Alharbi et al. 2024; Merchuk and Saranga 2013). Understanding WUE is crucial for improving plant sustainability in both managed and natural ecosystems.

### 2.9 Streamlining of Transpiration

Under drought stress, plants may exhibit structural modifications to reduce leaf surface area or alter leaf anatomy to minimize transpirational water loss. These adaptations are crucial for enhancing water conservation and improving survival in water‐limited environments. One common response is a reduction in leaf size, which directly decreases the total area available for transpiration, thereby lowering water loss. This phenomenon is often accompanied by an increase in leaf thickness, which can enhance water retention within the leaf tissue and improve structural integrity during periods of stress (Wyka et al. 2019).

In addition to size and thickness changes, plants may alter their leaf orientation to reduce direct exposure to sunlight and air movement, which can further limit transpiration rates. For instance, some species may adopt a more vertical leaf posture, minimizing the leaf area that is exposed to high temperatures and sunlight during the hottest parts of the day. This orientation can help reduce evaporative losses while still allowing for adequate light capture for photosynthesis (James and Bell 2000). Reduced stomatal density and size (Lawson and Leakey 2024; Sack et al. 2003), along with regulated aperture control (Karavolias et al. 2023), help balance CO_2_ uptake with water conservation. A thicker cuticle and trichomes form protective barriers (Goodwin and Jenks 2005), minimizing transpiration. Some species enhance mesophyll air spaces to store moisture efficiently (Yavas et al. 2023). These adaptations, combined with osmolyte accumulation and stress‐responsive genes, improve drought resilience and sustain photosynthesis, ensuring survival in arid environments (Qiao et al. 2024).

### 2.10 Antioxidant Defense to Counteract Oxidative Stress

Plants respond to water stress through multiple physiological and biochemical mechanisms aimed at mitigating damage and ensuring survival. One of the primary responses to drought conditions is the reduction of *g*_s_, which limits the entry of atmospheric CO_2_ into the chloroplast. This restriction slows or inhibits the Calvin cycle, preventing the regeneration of NADP^+^, a crucial electron acceptor in the electronic transport chain of chloroplasts. Consequently, excess electrons accumulate, particularly at the PSI level, which leads to the generation of ROS, such as O_2_·^−^ and H_2_O_2_ (Asada 1999). Additionally, drought stress has been linked to disruptions in Fe uptake by roots, resulting in increased Fe accumulation. This, in turn, could contribute to the production of ·OH^−^ via the Haber‐Weiss reaction, catalyzed by metal ions (Halliwell 2003; Price and Hendry 1991).

To counteract oxidative stress induced by drought, plants have developed a sophisticated antioxidant defense system comprising both enzymatic and non‐enzymatic components (Haghpanah et al. 2024; Noctor and Foyer 1998; Xu et al. 2024; Table 2). The enzymatic antioxidants include SOD, CAT, ascorbate peroxidase (APX), monodehydroascorbate reductase (MDHAR), glutathione reductase (GR), and peroxidase (POX), while non‐enzymatic antioxidants consist of ascorbate (ASC), glutathione (GSH), tocopherols, carotenoids, phenolic compounds, and proline (Pro; Oberoi 2019).

**TABLE 2:** Enzymatic and non‐enzymatic components to counteract oxidative stress induced by drought.

Name	Antiox type	Species	Drought tolerance effect	References
ASC‐GSH cycle	Enzymatic antioxidants	Plum (*Prunus* sp.)	Enhances drought adaptation by maintaining redox states	Diaz‐Vivancos et al. (2016) and Sofo et al. (2005)
CAT		Various	Prevents H_2_O_2_ leakage, mitigates oxidative stress	Mittler and Zilinskas (1994)
CAT and POX		Almond (*Prunus elaeagnifolia, P* *. webbi* )	Maintains stability during drought and recovery	Jurado‐Mañogil et al. (2024) and Martínez‐García et al. (2020)
Cu, Zn‐SOD		Tobacco ( *Nicotiana tabacum* )	Increased drought tolerance	Faize et al. (2011)
Cytosolic APX (cytapx)		Tobacco ( *N. tabacum* )	Enhanced WUE and photosynthetic rates	Faize et al. (2011)
Cytosolic APX (J8‐1 plum)		Plum (*Prunus* sp.)	Higher *A*_N_ and WUE under drought stress	Diaz‐Vivancos et al. (2016)
Glycolate oxidase		Various	Induces photorespiration to protect photosynthesis	Mittler and Zilinskas (1994)
GR		Plum (*Prunus* sp.)	Facilitates GSH recycling for redox maintenance	Diaz‐Vivancos et al. (2016)
MDHAR		Plum (*Prunus* sp.)	Facilitates ASC recycling for redox maintenance	Diaz‐Vivancos et al. (2016)
Carotenoids	Non‐Enzymatic antioxidants	Various	Prevent lipid peroxidation, protect photosynthesis	Munné‐Bosch and Alegre (2002) and Wujeska et al. (2013)
DHA accumulation		Cleopatra mandarin ( *Citrus reticulata* )	Increases drought susceptibility	Zandalinas et al. (2017)
Phenolic compounds		Olive ( *Olea europaea* )	Aid in water status regulation, prevent oxidative damage	Mechri et al. (2020)
Pro		Wheat ( *Triticum aestivum* )	Acts as osmoprotectant, improves drought tolerance	Per et al. (2017)
Tocopherols (α‐tocopherol)		Various	Stabilize membranes under drought	Munné‐Bosch and Alegre (2002) and Wujeska et al. (2013)

Abbreviations: antiox, antioxidant; AN, net photosynthetic rate; APX, ascorbate peroxidase; ASC, ascorbate; CAT, catalase; cytosolic APX (cytapx), cytosolic APX (J8‐1 plum line); GSH, glutathione; GR, glutathione reductase; H_2_O_2_, hydrogen peroxide; MDHAR, monodehydroascorbate reductase; POX, peroxidase; Pro, proline; SOD, superoxide dismutase; WUE, water use efficiency.

Given the increasing challenges posed by climate change, which include rising temperatures and declining water availability, the development and selection of drought‐tolerant plant genotypes are of paramount importance. Understanding the interplay between enzymatic and non‐enzymatic antioxidant responses will be crucial for breeding and engineering plants capable of withstanding future water‐deficient conditions.

### 2.11 Resurrection Plants: Mechanisms of Desiccation Tolerance

Resurrection plants like *Xerophyta viscosa*, *Craterostigma plantagineum* and *C. wilmsii*, *Sporobolus stapfianus* are vascular plants capable of surviving extreme dehydration, losing up to 95% of their water content and resuming normal function upon rehydration (Bartels 2005; Cooper and Farrant 2002; Li et al. 2009; Peters et al. 2007; Scott 2000). This desiccation tolerance is achieved through physiological, biochemical, and molecular adaptations that protect cellular structures during dehydration (Scott 2000).

Key biochemical mechanisms include the accumulation of LEA proteins and dehydrins, which stabilize membranes and proteins (Peters et al. 2007). Additionally, high concentrations of sugars such as trehalose, sucrose, and raffinose family oligosaccharides (RFOs), particularly raffinose, replace water molecules, forming a vitrified state that prevents mechanical stress (Peters et al. 2007). To mitigate oxidative stress, resurrection plants enhance antioxidant defenses by upregulating enzymes such as SOD and CAT, along with non‐enzymatic antioxidants such as flavonoids (Gupta et al. 2019).

Structural adaptations also contribute to desiccation tolerance. Many resurrection plants fold or curl their leaves to reduce water loss, while modifications in cell wall composition, such as increased lignin and suberin, prevent cell collapse (Shivaraj et al. 2018).

At the molecular level, these plants activate stress‐responsive TFs, such as dehydration‐responsive element‐binding proteins (DREBs) and ABA‐regulated genes, triggering protective responses. Genomic studies suggest that resurrection plants reactivated ancestral mechanisms found in desiccation‐tolerant seeds (Bartels 2005; Bartels and Hussain 2011; Shivaraj et al. 2018).

Understanding these mechanisms has significant implications for improving drought resistance in crops. By identifying key genes responsible for desiccation tolerance, researchers aim to enhance crop resilience to water scarcity through genetic engineering. As climate change exacerbates drought stress, resurrection plants offer valuable insights for sustainable agriculture and ecosystem conservation.

## 3 Chapter 2—Mechanisms and Approaches to Enhance Drought Tolerance: Molecular, Transcriptional, and Genetic Perspectives

The above‐mentioned physiological and morphological adaptations are accompanied by gene expression changes to help the plants survive in drought environments (Borràs et al. 2021). Presently, thanks to the boost of molecular biotechnology, gene expression studies are increasingly employed to study the response modulation at the “omics” level (You et al. 2019). These approaches could be very advantageous for breeders to define the characteristics of drought‐tolerant plants and develop tolerant crops.

In fact, genetic and breeding approaches have already enabled the development of water stress tolerant varieties through traditional selection, genetic engineering, and genome editing. Several traits that enhance plant survival in arid environments (higher WUE, bigger root depth, and osmotic regulation) have been identified and improved (Figure 1). The combination of strategies of advanced biotechnology with conventional breeding techniques is a sustainable solution for agricultural production in drought‐prone areas (Askari‐Khorasgani and Pessarakli 2021; Pradhan et al. 2024).

### 3.1 Molecular Mechanisms

Water stress triggers a molecular response in plants by which diverse signals are transmitted through multiple signaling pathways to regulate the expression of drought‐responsive genes and proteins (Kaur and Asthir 2017).

Drought and salinity stresses trigger ABA signalling, initiating a cascade in which sucrose‐nonfermenting‐1‐related protein kinases (SnRK2s) function upstream of TFs such as ABA‐INSENSITIVE 3 (ABI3), ABI5, and ABA‐responsive element‐binding factors (ABFs) to regulate LEA gene expression via ABA‐responsive elements (ABRE; Hsiao 2024). Initially, water stress induces a ROS increase, which acts as a stress‐sensing signal to activate signal transduction involving secondary messengers such as H_2_O_2_, Ca^2+^, and ABA (Cruz de Carvalho 2008). OSCA1, an osmosensitive ion channel, plays a crucial role in this process by mediating Ca^2+^ influx in response to osmotic stress, further amplifying the signal transduction pathway (Pei et al. 2022). This signal cascade subsequently activates mitogen‐activated and Ca^2+^‐dependent protein kinases (MAPKs and CDPKs), which, through phosphorylation or dephosphorylation, modulate the activity of TFs, including those involved in ABA signalling, thereby fine‐tuning the plant's stress response (Bashir et al. 2021).

The gene expression changes associated with this molecular response drive a reprogramming process at the onset of all plant levels, which determines the plant's tolerance or sensitivity to drought conditions (Padilla Herrero 2023).

#### 3.1.1 Transcription Factors (TFs)

Some TFs bind to specific sequences at the promoter or enhancer regions of stress‐responsive genes to modulate gene expression and control multiple downstream genes at the same time (Manna et al. 2021), which makes TFs interesting targets in plant breeding studies. Some TF families have been identified as key regulators in drought response, and their positive (+) or negative (−) contribution to water stress tolerance has been studied in several crops. The main TF families involved in drought responses are DREB (dehydration responsive element binding), bZIPs (basic leucine zipper), MYB superfamily, HSF (heat shock factors), NAC (nascent polypeptide‐associated complex), and WRKY (Table 3). Below, the roles of several TF families in drought tolerance are described.

**TABLE 3:** The main TF families involved in drought responses.

TFs	Function	Contribution in drought responses
DREB	Main regulators of abiotic stress tolerance in plants (Hussain et al. 2021; Manna et al. 2021).	Arabidopsis (Dubouzet et al. 2003; Haake et al. 2002; Sakuma et al. 2006, Sakuma et al. 2002; Wang et al. 2008), chickpea (Das et al. 2021), pepper (Padilla et al. 2023), rice (Berchembrock et al. 2022; Bihani et al. 2011; Oh et al. 2005; Wang et al. 2008), wheat (Kume et al. 2005; Pellegrineschi et al. 2004; Zhou et al. 2020)
bZIPs	An ABA‐dependent mechanism (Zong et al. 2016).	(+) Arabidopsis (Zhang, Zhang, Du, et al. 2015; Zhang, Zhang, Xia, et al. 2015), cotton (Liang et al. 2016), rice (Lu et al. 2009; Tang et al. 2012; Xiang et al. 2008; Yoon et al. 2017), sweet potato (Wang et al. 2019); (−) pepper (Joo et al. 2019), soybean (Zhang et al. 2020), tomato (Pan et al. 2017).
MYB	Water stress (Zhang et al. 2012) or oxidative stress mitigation response (Du et al. 2018; Wang, Chen, et al. 2017; Wang, Zhang, et al. 2017), among others.	Barley (Alexander et al. 2019), rice (Ithal and Reddy 2004), soybean (Du et al. 2018; Wang, Chen, et al. 2017; Wang, Zhang, et al. 2017), wheat (Mia et al. 2020).
NAC	Water stress responses usually by interacting with the promoter region of drought responsive genes (Bashir et al. 2021).	*Capsicum annuum* (Padilla Herrero 2023), rice (+) (Hong et al. 2016; Hu et al. 2006; Nakashima et al. 2007; Wang et al. 2020); (−) (Shen et al. 2017), tomato (Thirumalaikumar et al. 2018; Wang et al. 2016)
WRKY	Central regulators of abiotic stress response since they can control the regulation of multiple abiotic stresses at a time (Manna et al. 2021).	(+) Cotton (Chu et al. 2015; Li et al. 2021), maize (Cai et al. 2014), rice (Qiu and Yu 2009; Raineri et al. 2015), soybean (Wang et al. 2015; Zhou et al. 2008), sweet potato (Zhu et al. 2020), tomato (Chen et al. 2024; Gao et al. 2020); (−) Arabidopsis (Chen et al. 2017; Sun and Yu 2015), cotton (Jia et al. 2015; Liu et al. 2016; Yan et al. 2015), tomato (Ahammed et al. 2021).

Abbreviations: +/−, positive or negative contribution to water stress tolerance; bZIPs, basic leucine zipper; DREB, dehydration responsive element binding; HSF, heat shock factors; NAC, nascent polypeptide‐associated complex; TFs, transcription factors; WRKY, Worky.

#### 3.1.2 Drought‐Responsive Proteins: Aquaporins and LEA Proteins

Within the transcriptomic reprogramming that plants undergo under drought stress, we find the regulation of genes coding for drought‐responsive proteins to achieve drought tolerance (Kumar et al. 2021). LEA proteins, which are associated with seed desiccation tolerance, are drought‐responsive proteins, and their accumulation has been directly linked with the protection of cell membranes from denaturation, among other functions (Aslam et al. 2015; Park et al. 2003). Dehydrins are included in group 2 of LEA proteins, and they have been associated, in multiple species, with water stress tolerance through their chaperone function, ability to perform osmotic adjustments, and ROS scavenging properties (Hara et al. 2013; Riyazuddin et al. 2022). Hydrophilins are desiccation‐resistance proteins that are able to protect enzymatic activities from water‐loss effects in a wide range of water potentials (Ψ_w_; Holehouse and Kragelund 2024; Reyes et al. 2005).

Aquaporins are integral membrane proteins essential for transporting water and small solutes across biological membranes, playing a crucial role in regulating plant water status and adapting to drought stress (Afzal et al. 2016; Bárzana and Carvajal 2020). Aquaporins are sensitive to changes in Ca^2+^ concentration, which are regulated by ABA signalling to increase WUE in drought conditions, specifically in guard cells for the control of stomatal aperture (Hong‐Bo et al. 2008; Shao et al. 2008). By controlling water flux in different tissues, aquaporins help maintain Ψ_P_ and overall hydration, thereby enhancing drought tolerance (Ni et al. 2024; Shivaraj et al. 2021). The expression and activity of aquaporins are tightly regulated in response to drought stress and developmental signals through transcriptional and post‐translational modifications. The functional diversity of aquaporins, with various isoforms serving specific roles in water absorption and redistribution, enables plants to optimize water use, particularly under drought conditions. Harnessing this knowledge through biotechnological approaches, such as genetic engineering and gene editing (e.g., the Clustered Regularly Interspaced Short Palindromic Repeats and associated Cas proteins, CRISPR/Cas9), offers opportunities to improve drought resistance in crops, enhancing their WUE and mitigating the adverse effects of drought on agricultural productivity (Martinez‐Ballesta and Carvajal 2014; Patel and Mishra 2021; Rabeh et al. 2024). Park et al. (2005) introduced a LEA protein gene from rape plants (*ME‐leaN4*) into lettuce, which improved growth in drought conditions in the transgenic plants. Cheng et al. (2002) improved drought tolerance in transgenic rice plants by the introduction of dehydrin genes from wheat (*PMA80* and *PMA1959*). Sade et al. (2009) obtained bigger yields and more productivity under drought conditions by the overexpression of the aquaporin gene *SlTIP2‐2* in tomato transgenic plants, related to sustained stomatal opening and transpiration.

#### 3.1.3 Long‐Distance Signaling mRNAs

Signalling in plants under drought stress comprises a long‐distance signal transmission through multiple tissues and organs involving mobile messenger RNAs (mRNAs), small proteins, peptides, and metabolites, among other molecules (Heeney and Frank 2023; Takahashi and Shinozaki 2019). Particularly, mobile mRNAs have been identified as drought tolerance mediators in cucumber grafted onto pumpkin (Davoudi et al. 2022), grafted grapevine (Pagliarani et al. 2017), and grafted pear (Hao et al. 2020). The authors identified mRNAs that moved from the rootstock to the scion as a response to drought stress to increase tolerance in the grafted plants.

### 3.2 Genetic and Breeding Approaches

#### 3.2.1 Breeding for Drought Tolerance

##### 3.2.1.1 Challenges of Breeding for Drought Tolerance

**FIGURE 2:**
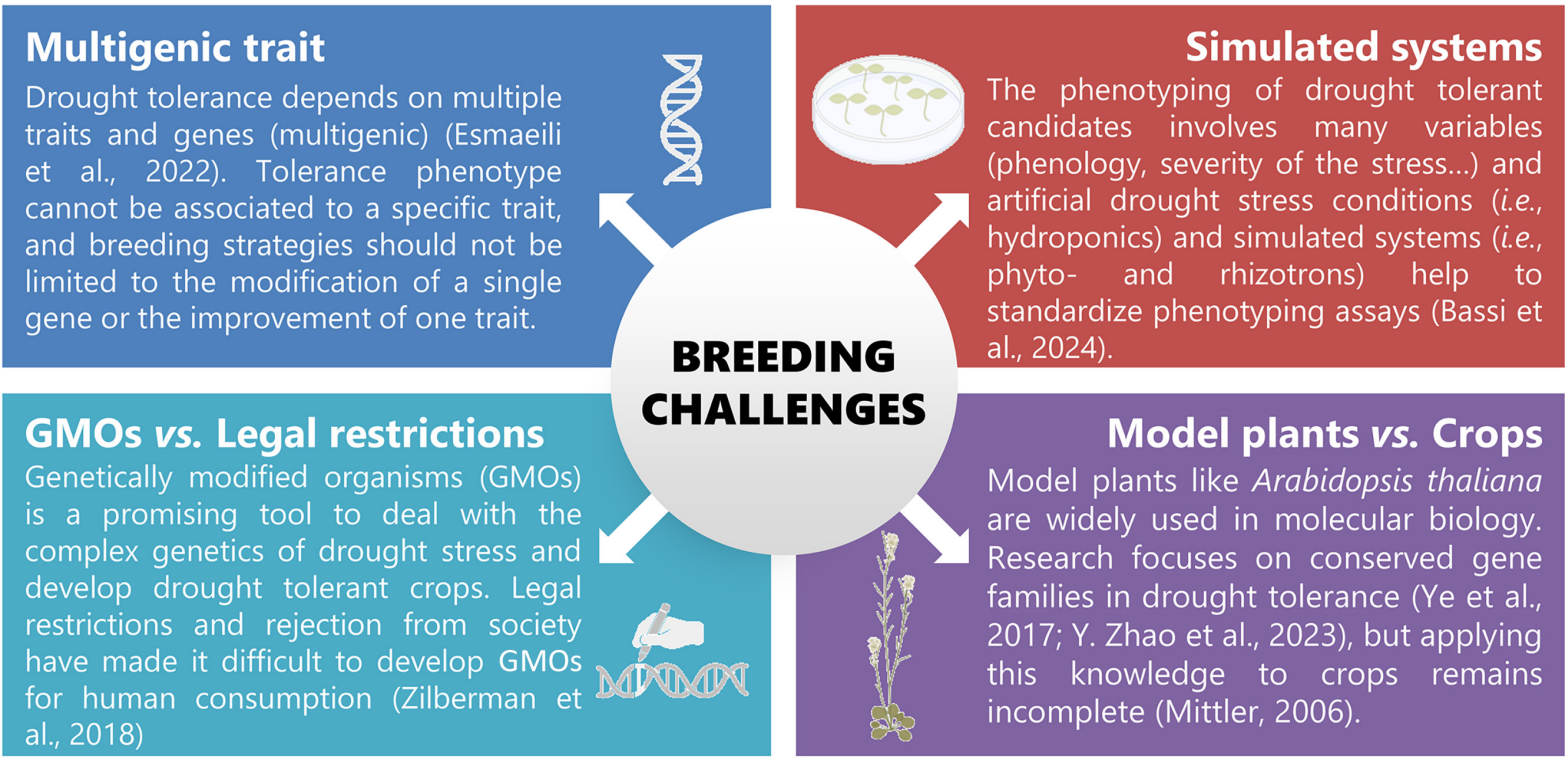
Challenges associated with the complexity of abiotic stresses and the breeding for drought tolerance. *Source:* (Bassi et al. 2024; Esmaeili et al. 2022; Mittler 2006; Ye et al. 2017; Zhao, Gao, An, et al. 2023; Zhao, Duan, et al. 2023; Zilberman et al. 2018). Most elements of this scheme were created with BioRender.com (CC‐BY 4.0 license).

Water stress tolerance is a highly demanded trait in modern breeding programs, as drought events as a consequence of climate change are only expected to increase in the near future. Growers need drought‐tolerant crops able to grow under water scarcity conditions to avoid high yield losses and ensure food supplies on a global scale. However, breeding for drought tolerance implies several challenges associated with the complexity of abiotic stresses (Figure 2).

##### 3.2.1.2 The Starting Point for Breeding Programs

Identifying more efficient and resilient crops is the most suitable, though challenging, solution to overcome different stresses (Solh and van Ginkel 2014). Genetic diversity is the starting point for any breeding program; without diversity, plant breeding could not be employed to improve the target crop (Bassi et al. 2024; Song, Ong, et al. 2024; Song, Han, et al. 2024). Crop domestication and the Green Revolution, along with industry‐designed and consumer‐designed modern varieties, are responsible for the narrow diversity in cultivated crops (Folta and Klee 2016; Negri et al. 2009; Ray et al. 2019). In fact, it is estimated that 75% of biodiversity in the main crops has been lost in the last decades (Food and Agriculture Organization 2016; Raggi et al. 2021; Ramirez‐Villegas et al. 2022).

Genetic resources such as landraces and wild relatives of crops can be used in breeding programs, as they store diversity to a greater extent than modern cultivars (Barba‐Espín and Acosta‐Motos, 2022; Cubero 2013; Galmes et al. 2013; Ibañez et al. 2021; Plazas et al. 2022). These genetic resources have been used in drought tolerance because most of them are adapted to stressful environments and preserve favorable genetic combinations for tolerance‐related traits (Muñoz‐Perea et al. 2007; Rosero et al. 2020). In fact, a breeder can cross the variety to be improved with other genetic material with desirable traits from different origins or traditional varieties, which represent a population of individuals with an expected significant variability. Once variability is obtained, key characteristics are identified, and the selection of desirable traits is performed.

It is crucial to understand that the genetic component (G) interacts with the environment (E) in which plants are cultivated, resulting in the observed phenotypes (*P* = G × E; Cubero 2013). This interaction highlights the importance of considering the environmental component in the development of breeding programs. The environment can significantly influence the phenotype, often being responsible for the observable traits due to the adaptation of the variety to specific environmental conditions (Salekdeh et al. 2009). Therefore, breeding programs must account for both genetic diversity and environmental factors to effectively select and develop varieties with optimal performance and resilience in varying conditions. This holistic approach ensures the development of robust varieties that can thrive under diverse environmental stresses, including water stress (Arab et al. 2023; Vadez et al. 2013). In this regard, appropriate phenotyping is essential to identify genotypes adapted to drought (Reynolds et al. 2020).

##### 3.2.1.3 Validation of Drought Tolerant Varieties

When validating plant varieties for water stress tolerance, it is essential to conduct trials under the most realistic possible drought conditions (Flores‐Saavedra et al. 2023). The quantitative nature of traits related to WD, along with the variability in the duration and intensity of water stresses across different plant phenological stages, environments, and crops, has hindered the development of consensus and universal protocols for comprehensive phenotyping of candidate genotypes (Passioura 2012).

Methods to induce WD to evaluate plant responses are very diverse. In field or greenhouse assays, WD is often induced by withholding irrigation, simulating drought (Akbudak et al. 2020; Plazas et al. 2019); by reducing irrigation to a certain percentage of field capacity (Azizi et al. 2021; Gisbert‐Mullor et al. 2023; Penella, Calatayud, et al. 2017), or by reducing evapotranspiration percentage (Galmes et al. 2013; Padilla et al. 2021; Semida, Abdelkhalik, et al. 2021).

To accurately assess water stress tolerance, trials should be performed over several years and across multiple locations to account for genotype‐environment interactions. Replicating drought conditions in controlled environments, such as laboratories and greenhouses, often introduces errors, as these settings fail to capture the full spectrum of environmental interactions present in actual field conditions (Mahalingam 2015). Therefore, transferring findings from controlled conditions to real‐world environmental stress scenarios is crucial.

In the field, plants are frequently exposed to a combination of abiotic and biotic stresses, making it necessary to conduct trials in the actual cultivation areas. Multi‐location trials are critical for observing how genotypes interact with different environments (Penella, Nebauer, et al. 2017). These trials should be repeated over several years to confirm the improved variety's tolerance by studying yield and fruit quality.

Recently, the use of rhizotrons, hydroponics, climatic chambers, in vitro culture, and other simulated systems has gained considerable attention for studying plant responses to water stress (Dutta et al. 2023), in many cases using an osmotic agent, such as polyethylene glycol (Liu et al. 2021; López‐Serrano et al. 2019; Penella et al. 2014). However, their validation for plant breeding purposes is still ongoing. Continuous efforts are needed to bridge the gap between controlled environment studies and practical field applications to enhance the effectiveness of breeding programs aimed at developing water stress‐tolerant varieties (Flores‐Saavedra et al. 2023). These efforts include the integration of advanced phenotyping technologies, the development of better simulation models, and the increased collaboration between researchers and practitioners to ensure that laboratory findings translate effectively into field success.

#### 3.2.2 Breeding Strategies

##### 3.2.2.1 Conventional Versus Speed Breeding

Conventional breeding is a time‐consuming strategy that takes several years to find the right parents by phenotyping and making crosses to obtain the desired varieties. Most conventional breeding programs have based their selection on productive traits such as the seed yield, which could be influenced by the environment and management practices, and thus it should not be the principal trait in the selection process for drought tolerance (Varshney et al. 2021). As mentioned before, drought tolerance is a combination of many traits, and conventional breeding techniques are time and effort limited. Recently, speed breeding has emerged as a complementary strategy for conventional breeding, by which crop development is sped up to reduce the time between crop cycles. While conventional breeding usually takes from 8 to 10 years to raise a new cultivar, speed breeding could produce up to five or six generations annually. For this, plants are grown in controlled environments with respect to temperature, humidity, and photoperiod, and several generations can be raised in a year (Raza et al. 2023). The controlled growth conditions allow shortening the growth cycle and harvesting several generations in a single cycle, which reduces the duration of new varieties development. Moreover, speed breeding could be combined with modern “omics” technologies and genome‐editing tools to advance breeding programs towards a more efficient and smart breeding (Raza et al. 2023). The controlled growth conditions allow shortening the growth cycle and harvesting several generations in a single cycle, which reduces the duration of new varieties development. Moreover, speed breeding could be combined with modern “omics” technologies and genome‐editing tools to advance breeding programs towards a more efficient and smart breeding.

##### 3.2.2.2 Omics‐Assisted Breeding Techniques

More recently, new “omics” tools can help breeders accelerate the breeding process, as they are high‐throughput technologies that increase the efficiency of the process, reducing time, economic resources, and human resources (Rosero et al. 2020). Some of these strategies include the genome‐wide association study (GWAS), quantitative trait loci (QTL) mapping, or marker‐assisted selection (MAS).

GWAS is based on the association of genetic variation to markers throughout the genome, and it is used to associate specific regions to the observed variation in a trait through the combination of genomic and phenotypic information and statistical analysis (Alqudah et al. 2020). QTL mapping is used to identify genomic regions related to the drought tolerance phenotype, and to define candidate genes or regions linked to tolerance traits (Raza et al. 2023). These identified candidates are employed in MAS to screen and select the best individuals by genotype, which shortens the selection process as compared to phenotypic screening (Kevei et al. 2024; Thompson et al. 2017; Check more examples in Table 4).

**TABLE 4:** Omics‐assisted breeding techniques for drought stress tolerance in crops.

Crop	Breeding technique	Key achievements	References
Chickpea	QTL introgression	Enhanced drought tolerance through QTL introgression	Bharadwaj et al. (2021) and Varshney et al. (2014), Varshney et al. 2013)
Maize	MAS	Improved drought tolerance using marker‐assisted selection	Beyene et al. (2016)
Rice	QTL introgression, MAS	Developed drought‐tolerant varieties using QTLs for grain yield under drought stress	Shamsudin et al. (2016)
	Breeding programs (IRRI, India, Nepal)	Developed high‐yield, drought‐tolerant varieties through QTL identification and gene introgression	Khan et al. (2020) and Sandhu et al. (2019)
	QTL introgression (root system focus)	Introduced QTL controlling root architecture for deeper roots and higher yield under drought	Uga et al. (2013)
Wheat	QTL identification and introgression	QTLs from wild relatives introgressed to improve drought tolerance	Merchuk‐Ovnat et al. (2016)

Abbreviations: MAS, marked‐assisted selection; QTL, quantitative trait loci.

##### 3.2.2.3 Genome‐Editing Approaches

Genome editing techniques avoid the need for natural genetic diversity, and they have a vast potential for crop modification to achieve drought tolerance. However, as explained before, GMOs are highly restricted for human consumption. In the last decades, several genes related to tolerance to various abiotic stresses have been identified, and thus these candidate genes can be exploited by genetic engineering to increase the drought tolerance of crops (Kumar et al. 2021; Manna et al. 2021).

Several transgenic tolerant plants have been developed by transferring genes from different crops (horizontal transfer) or by engineering genes in the same cultivar. Some of these transgenic plants modified by overexpression or silencing of drought‐related TFs can be found in section 1.1. Other transgenic plants have been engineered for higher osmoprotectant sugar content (Garg et al. 2002; Karim et al. 2007; Li et al. 2011), or show an increased proline content after the modification of dehydrin genes (Bao et al. 2017; Brini et al. 2007; Chiappetta et al. 2015; Liu et al. 2015), leading to drought stress tolerance.

Ultimately, gene‐editing methods that rely on the modification of a few nucleotides have been considered separately from the widely known transgenic modifications, as the scientific community has made efforts to obtain specific regulations for this technique (Bassi et al. 2024). The CRISPR/Cas9 system is an interesting opportunity for researchers, as it can be used to edit genes, single bases, or perform prime editing in multiple crop plants (Raza et al. 2023). In this way, both positive and negative players in drought tolerance can be either modified or knocked out to obtain drought‐tolerant plants (Table 5).

**TABLE 5:** CRISPR/Cas9 gene‐editing strategies for enhancing drought tolerance in crops.

Gene‐editing approach	Crop	Target gene	Modification & outcome	References
Loss‐of‐function	Tomato	*SlMAPK3*, *SlNPR1*	Reduced drought tolerance, increased oxidative damage (*slnpr1* mutants), altered stomatal behavior, down‐regulated drought‐responsive genes	Li et al. (2019) and Wang, Chen, et al. (2017), and Wang, Zhang, et al. (2017)
Gene editing	Maize	*ARGOS8*	Novel variants with improved drought tolerance and higher yield under flowering‐stage drought stress	Shi et al. (2017)
Stomatal regulation	Arabidopsis	*OST2*	Mutants exhibited drought tolerance via reduced stomatal aperture; applicability in crops depends on fruit yield impact	Osakabe et al. (2016)
Stomatal regulation	Rice	*SRL1/2*	Lower *g*_s_, fewer stomata, curled leaves, increased panicle and grain yield‐potential for drought‐tolerant breeding	Liao et al. (2019)

Abbreviation: *g*_s_, stomatal conductance.

## 4 Chapter 3—Plant Nutrition, Trendy Beneficial Nutrients, and Biofortification

Nutrition plays a crucial role in the response of plants to drought stress, as it directly influences their physiological and biochemical processes, growth, and productivity (Iqbal et al. 2020). During drought conditions, plants experience alterations in nutrient uptake, transport, allocation, and utilization, which can profoundly impact their resilience and ability to withstand water scarcity (Taiz and Zeiger 1991, 2002; Waraich et al. 2011). Therefore, it is essential to consider various aspects of nutrition to effectively manage and mitigate the detrimental effects of drought stress on crop performance. Here are some key factors to consider regarding nutrition and drought:

Drought stress significantly impacts plant nutrition by reducing water availability, which in turn limits the diffusion of nutrients to plant roots (Taiz and Zeiger 2002; Taiz and Zeiger 1991; Waraich et al. 2011). As soil moisture decreases, the mobility of nutrients declines, thereby impairing their uptake and assimilation by plants. Moreover, drought‐induced changes in soil pH, microbial activity, and root exudation further affect nutrient availability, often exacerbating nutrient imbalances. These imbalances, especially deficiencies in essential macronutrients such as nitrogen (N), phosphorus (P), and potassium (K, K^+^), can impair vital physiological processes, reducing photosynthesis, growth, and overall plant productivity (Nieves‐Cordones et al. 2019; Seleiman et al. 2021). N is a critical component of proteins, nucleic acids, and many other important molecules in plant cells. Phosphorus (P) is necessary for energy transfer and cell division. Sulphate (S) is critical to construct cysteine, methionine, and glutathione (GSH). K^+^ is involved in the regulation of water balance and many other cellular processes. Ca^2+^ and magnesium (Mg, Mg^2+^) are important structural components of plant cells (Hawkesford et al. 2012; Kumari et al. 2024; Li et al. 2020; Maathuis 2009). Micronutrient deficiencies, including those of iron (Fe), zinc (Zn), and manganese (Mn), also disrupt enzymatic functions critical for stress tolerance, further diminishing the plant's ability to cope with drought (Seleiman et al. 2021).

Proper nutrition is essential for plants to adapt to drought conditions, as nutrients play a key role in osmotic adjustments and ion homeostasis. Osmolytes such as soluble sugars, proline, and amino acids accumulate in plant cells to help regulate Ψ_π_, protecting against cellular dehydration. Essential ions, particularly K^+^ and Ca^2+^, are crucial for maintaining cell turgor, membrane stability, and enzyme activity, all of which contribute to sustaining plant growth and metabolic function under water stress (Gimeno et al. 2014; Nieves‐Cordones et al. 2019; Ozturk et al. 2021). In addition, maintaining an adequate supply of antioxidant nutrients, such as vitamins C and E, and minerals such as selenium (Se) and copper (Cu), helps plants mitigate oxidative damage caused by drought‐induced ROS. These antioxidants enhance the plant's ability to manage oxidative stress and improve overall resilience to drought (Nieves‐Cordones et al. 2019; Seleiman et al. 2021; Waraich et al. 2011).

To optimize plant nutrition during drought stress, nutrient management strategies must be tailored to water‐limited conditions. This includes adjusting fertilizer rates, timing, and application methods to improve nutrient efficiency and minimize losses. Techniques such as using water‐soluble or slow‐release fertilizers, and applying foliar nutrient sprays, can help maintain nutrient availability and support optimal plant development during drought. By integrating these approaches, farmers can mitigate nutrient deficiencies and bolster plant tolerance to water scarcity, improving crop productivity in drought‐prone regions and contributing to sustainable agriculture (Cramer et al. 2008; Singh et al. 2022; Trenkel 2010).

In modern agriculture, the quest to enhance crop resilience to drought has increasingly focused on the role of specific nutrients that help mitigate the effects of water stress. These essential nutrients not only support plant growth and productivity during drought conditions, but also improve WUE, reinforce root systems, and strengthen overall crop health (Cakmak et al. 2022; Ma, Zhao, et al. 2023; Ma, Yu, et al. 2023). By optimizing nutrient availability, plants can better manage WD and sustain growth under challenging environmental conditions (Ashraf 2010; Broadley et al. 2012; Moulick et al. 2024).

**FIGURE 3:**
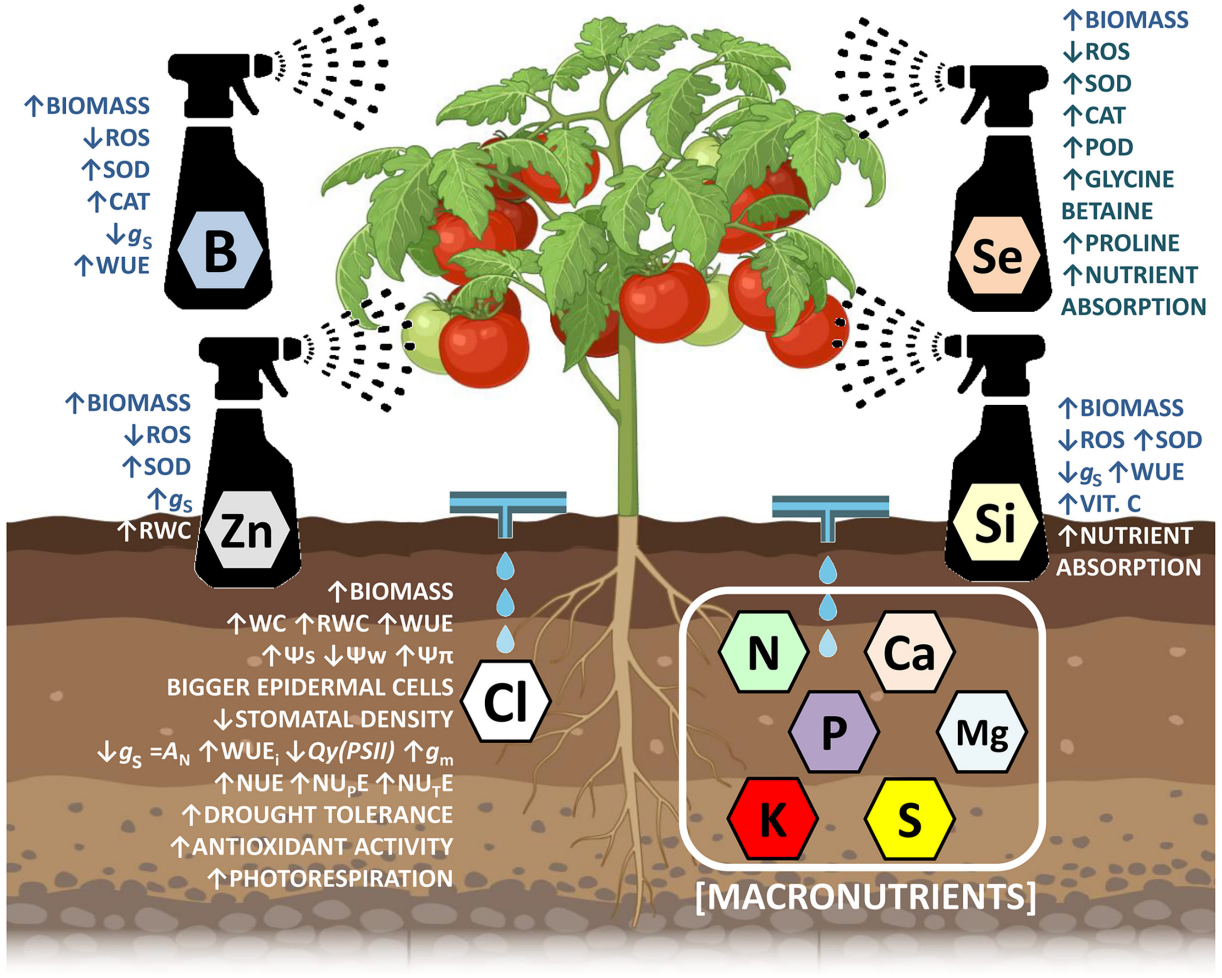
Scheme of main macronutrients and trendy beneficial nutrients (Chapter 3, Plant nutrition, trendy beneficial nutrients, and biofortification). (macronutrients) Ca, Ca^2+^, Calcium; Mg, Mg^2+^, magnesium; N, N$O_{3}^{-},$nitrogen/nitrate; P, P$O_{4}^{2-},$phosphorus/phosphate; S, S$O_{4}^{3-},$sulphur/sulphate (Hawkesford et al. 2012; Kudoyarova et al. 2015; Kumari et al. 2024; Maathuis 2009; Waraich et al. 2011); (micro and beneficial macronutrient) Cl, Cl^−^, chloride (Colmenero‐Flores et al. 2019; Franco‐Navarro et al. 2021; Franco‐Navarro et al. 2019; Franco‐Navarro et al. 2016; Lucas et al. 2024; Peinado‐Torrubia et al. 2023; Rosales et al. 2020a; Rosales et al. 2020b); (biofortification with beneficial nutrients) B, H_3_BO_3_/[B(OH)_4_]^−^, boron (Haque 2024; Ramirez‐Builes et al. 2024); Se, [SeO_3_]^2−^, selenium (Abdalla et al. 2024; Moulick et al. 2024); Si, [SiO_3_]^2−^, silicon (Abdalla et al. 2024; Irfan et al. 2023); Zn, Zn^2+^, zinc (Semida, Abdelkhalik, et al. 2021; Stanton et al. 2022); CAT, catalase; WUE, WUEi, intrinsic; *g*_m_, mesophyll conductance to CO_2_ diffusion; *A*_N_, net photosynthetic rate; NUE, nitrogen use efficiency; Qy(PSII), quantum yield, PSII efficiency; RWC, relative water content; ROS, reactive oxygen species; *g*_S_, stomatal conductance; SOD, superoxide dismutase; WC, water content; WUE, water‐use efficiency. *Source:* Most elements of this scheme were created with BioRender.com (CC‐BY 4.0 license).

A promising strategy within this context is biofortification, which involves increasing the concentration of essential nutrients in crops through breeding, agronomic practices, or genetic engineering (Broadley et al. 2012; Rehman et al. 2021). Biofortification not only improves plant resilience to stress but also addresses micronutrient deficiencies in human diets, making it a dual‐benefit approach. By fortifying crops with key nutrients such as Zn, Fe, and Se (Figure 3; Abdalla et al. 2024; Broadley et al. 2012; Mishra et al. 2022; Rehman et al. 2021; Stanton et al. 2022), biofortification can enhance the plants' natural defense mechanisms, such as boosting antioxidant activity and improving cellular water retention. As a result, biofortified crops are better equipped to withstand drought while at the same time providing nutritionally rich food to address global malnutrition challenges. This integrative approach underscores the growing importance of targeted nutrient management in both the improvement of agricultural sustainability and the enhancement of human health (Hamdy et al. 2003; Hatfield et al. 2011; Mishra et al. 2022).

### 4.1 Boron (B)

Boron (B) plays a crucial role in plant tolerance to drought stress by modulating key physiological and biochemical processes (Haque 2024; Figure 3). One of its primary functions is maintaining cell wall integrity through the cross‐linking of pectic polysaccharides, particularly rhamnogalacturonan‐II, which stabilizes cell walls and preserves cellular structure during WD conditions (Hays et al. 2024). Under drought stress, plants experience increased ROS production, leading to oxidative damage. B mitigates this by enhancing the activity of antioxidant enzymes such as SOD and CAT, which scavenge ROS and reduce oxidative damage (Abdel‐Motagally and El‐Zohri 2018; Aydin et al. 2019). This regulation of oxidative stress contributes to cellular homeostasis, helping plants maintain growth and metabolic activity even under limited water availability.

In addition to its structural and antioxidative roles, B influences the expression of drought‐responsive genes involved in water transport and stress signalling pathways. B regulates aquaporins, proteins responsible for facilitating water movement across cell membranes, which enhance water uptake and distribution in plants under drought conditions (Nicolas‐Espinosa et al. 2024). Furthermore, B interacts with phytohormones such as ABA to modulate stomatal behaviour, promoting stomatal closure to minimize water loss through transpiration. This multifaceted role of B in drought tolerance underscores its importance in maintaining cellular function and supporting plant survival during water stress (Qu et al. 2024).

### 4.2 Zinc (Zn)

Zn application to crops (generally as a foliar spray) plays a pivotal role in enhancing plant resilience to drought stress (Figure 3). One of the earliest and most sensitive stages to drought stress is seed germination, which directly affects leaf density and overall productivity (Hassan et al. 2020). Zn application, particularly through seed priming, has been shown to improve germination and early seedling growth in crops such as maize, wheat, and chickpea by boosting the synthesis of growth hormones such as indole‐3‐acetic acid (IAA) and gibberellic acid (GA3). These hormones promote longer plumule development and increase seedling dry weight, even under WD conditions. Additionally, Zn is a vital cofactor for numerous enzymes involved in key metabolic processes, such as RNA polymerase, carbonic anhydrase, and Cu/Zn‐SOD. These enzymes support transcription, photosynthesis, and ROS detoxification processes, helping plants maintain metabolic stability and protect cellular structures from oxidative damage during drought (Hassan et al. 2020).

Beyond germination, Zn significantly enhances drought tolerance by improving membrane stability, water relations, and antioxidant defense. An adequate Zn supply helps maintain cell membrane integrity by reducing electrolyte leakage and improving the relative water content (RWC) of plant tissues, which is a critical indicator of drought tolerance (Krebs et al. 2010; Semida, Abdelkhalik, et al. 2021; Waraich et al. 2011). Zn also supports photosynthetic efficiency by increasing chlorophyll and carotenoid content, promoting larger leaf areas and higher *g*_s_, which contribute to better WUE (Karim et al. 2012). Furthermore, Zn enhances the antioxidant activity of enzymes, such as SOD and CAT, mitigating oxidative damage from ROS generated during drought stress (Stanton et al. 2022). This multifaceted role of Zn, from improving water retention and leaf water status to stabilizing cellular structures and enhancing metabolic activity, makes it a key nutrient in helping plants adapt to and survive under water‐limited conditions.

### 4.3 Selenium (Se)

Se plays a crucial role in enhancing plant resilience to drought stress (Figure 3). Under drought conditions, plants treated with Se experience a significant reduction in ROS such as H_2_O_2_, which limits oxidative damage in plant cells. This results in improved antioxidant activity, particularly with increased levels of SOD, peroxidase (POD), and CAT (Lanza and Reis 2021; Walaa et al. 2010). Additionally, Se promotes the accumulation of essential osmoprotectants such as glycine betaine and proline, helping the plant retain water and maintain Ψ_P_, which is critical for drought tolerance (Semida, Abd El‐Mageed, et al. 2021).

Moreover, Se boosts nutrient uptake, particularly Ca^2+^, K^+^, and Sodium (Na^+^) ions, which are vital for maintaining cellular homeostasis under water stress (Hossain et al. 2021). Se‐treated plants also show an enhanced photosynthetic efficiency, improved growth, and elevated levels of flavonoids, anthocyanins, and phenolic compounds, all of which contribute to their ability to withstand drought (Walaa et al. 2010). As a result, Se nutrition not only mitigates the adverse effects of drought, but also promotes overall plant health and productivity in challenging environmental conditions.

### 4.4 Silicon (Si)

Silicon (Si) nutrition significantly enhances drought tolerance in plants by reinforcing both physiological and structural defenses (Figure 3). One of the primary benefits of Si is its ability to improve WUE by reducing leaf transpiration rates and facilitating better water uptake and transport in plants. Si accumulates in plant tissues, forming a protective layer that reduces water loss, thereby helping plants conserve moisture during periods of limited water availability (Hu et al. 2022; Khan, Awan, et al. 2023; Khan, Liu, et al. 2023; Khan, Liu, et al. 2023; Waraich et al. 2011). Additionally, Si plays a key role in improving the activity of antioxidant enzymes and the accumulation of Vitamin C, which helps mitigate oxidative stress caused by drought‐induced ROS, protecting cellular structures such as the chloroplasts (Hu et al. 2022; Waraich et al. 2011).

Si also promotes solute accumulation in plant cells, maintaining cell turgor and enabling physiological functions at lower Ψ_w_. In crops such as maize, sorghum, and cotton, Si has been shown to alleviate drought stress by enhancing root and shoot growth, maintaining leaf water content, and improving overall photosynthetic performance (Farooq et al. 2009; Maurel et al. 2015; Sonobe et al. 2011). Its ability to stabilize cell membranes and improve nutrient absorption further contributes to the plants' ability to withstand drought, making Si a vital element in sustainable agriculture under water‐deficit conditions (Ahmed and Fayyaz‐ul‐Hassen 2011). Its combination with Se is frequent due to its greater benefits for plants under drought stress (Abdalla et al. 2024; Hossain and Islam 2021; Hu et al. 2022; Seleiman et al. 2021).

### 4.5 Chloride (Cl^−^) as a Beneficial Macronutrient Against Drought Stress

Cl^−^ has been recently defined as a beneficial macronutrient (Cakmak et al. 2022; Colmenero‐Flores et al. 2019; Franco‐Navarro et al. 2016; Ma, Zhao, et al. 2023; Ma, Yu, et al. 2023) with specific roles that result in a higher WUE (Franco‐Navarro et al. 2016), N‐use efficiency (NUE; Rosales et al. 2020a; Rosales et al. 2020b), and higher efficiency in the assimilation of CO_2_ (Franco‐Navarro et al. 2019) in well‐watered glycophyte plants. All of those advantages have a positive influence on plant growth and development, particularly under drought conditions, promoting drought tolerance and resistance (Franco‐Navarro et al. 2021; Figure 3; Figure S2). Recently, Peinado‐Torrubia et al. (2023) reported that Cl^−^ stimulated photorespiration in plants treated with it, resulting in a reduced glycine/serine (Gly/Ser) ratio and increased formation of N$H_{4}^{+}$, CO_2_, and NADPH in the mitochondria, and Lucas et al. (2024) showed that Cl^−^ enhanced antioxidant activity and N metabolism in plants, helping them adapt to N deficiency.

#### 4.5.1 Challenging Traditional Misconceptions: Toxicity, Nutrient Imbalance, and Yield Reduction

While Cl^−^ as an essential micronutrient is mandatory in trace amounts for plant growth (Broyer et al. 1954; Colmenero‐Flores et al. 2019; Johnson et al. 1957; White and Broadley 2001), excessive Cl^−^ and Na^+^ accumulation in a context of salinity can be detrimental to plant health, leading to toxicity symptoms, nutrient imbalance and growth inhibition (Wang et al. 1989). As Paracelsus stated “The dose is the poison” (Grandjean 2016).

Sodium chloride (NaCl) is the most abundant soluble salt worldwide and has the strongest influence on soil salinity (Szabolcs 1989). It promotes the excessive uptake of Cl^−^ and other anions by plants, leading to symptoms such as leaf burn, chlorosis, and necrosis (Slabu et al. 2009; Tavakkoli et al. 2010). This stress ultimately reduces crop productivity (Geilfus 2019; Geilfus 2018a; Teakle and Tyerman 2010), particularly in salt‐sensitive species and fruit trees, including avocado (Bar et al. 1997; Lahav et al. 1993; Platt 1990).

In this context, Cl^−^ has traditionally been considered a toxic anion rather than a mineral nutrient for plants. This is a consequence of several reasons and misconceptions: (i) frequently, the function of Cl^−^ has not been adequately differentiated from that of its companion cations, mainly Na^+^ (Armengaud et al. 2004; Benlloch‐Gonzalez et al. 2008; Flowers 1988); (ii) the toxicity resulting from the excessive accumulation of Cl^−^ in sensitive organs in the context of salt stress and halophyte plants (Bell et al. 1997; Engel et al. 1994; Huang et al. 1995; Robinson 1986; Wang et al. 1989); and (iii) the widespread belief that Cl^−^ and N$O_{3}^{-}$ are two antagonistic molecules that compete for entry through the root and transport through the plant (Fricke et al. 1994a, 1994b).

In general, Cl^−^ concentrations higher than 20 mM in soil or an irrigation medium can cause toxicity in sensitive plant species, while in tolerant species, the Cl^−^ concentration can be four to five times higher without reducing their growth (Brumós et al. 2010). Differences in Cl^−^ toxicity concentrations are mainly related to differences in the sensitivity of leaf tissue to high Cl^−^ concentrations (White and Broadley 2001; Xu et al. 2000; Table S1).

#### 4.5.2 Change in Paradigm: Cl^−^ as a Beneficial Macronutrient That Improves Drought Resistance

Cl^−^ provides agronomic benefits beyond its role in salinity stress. It functions as both a micronutrient and a beneficial macronutrient under well‐irrigated and drought conditions. Cl^−^ is essential for several physiological processes in plants. When abundant, it accumulates in leaves at concentrations comparable to macronutrients such as K^+^ or N$O_{3}^{-}$ (Broyer et al. 1954; Cakmak et al. 2022; Colmenero‐Flores et al. 2019; Franco‐Navarro et al. 2016). This accumulation supports multiple physiological functions, highlighting its dual role as both a micronutrient and a beneficial macronutrient (Figure S2).

Leaf Cl^−^ accumulation promotes biomass production, improves leaf water status, and induces anatomical changes leading to larger leaf cells with higher water content (Franco‐Navarro et al. 2016). Cl^−^‐enriched plants exhibit reduced transpiration (lower *g*_s_) due to lower stomatal density, leading to significant water savings without compromising *A*_N_ since it is compensated by increased mesophyll conductance (*g*_m_; Franco‐Navarro et al. 2019). As a result, Cl^−^ nutrition enhances water‐use efficiency (WUE) and nitrogen‐use efficiency (NUE; Rosales et al., 2020), reducing water consumption while maintaining growth. Besides this, Cl^−^ improves drought resistance through (i) WD avoidance via increased WUE, water savings, and higher leaf water content and (ii) enhanced drought tolerance through osmotic adjustments that maintain leaf turgor and prevent dehydration (Franco‐Navarro et al. 2021).

Given agriculture's high‐water demand and climate change‐driven drought stress, increasing WUE is critical for crop productivity. A fertilization strategy that guarantees the presence of Cl^−^ at macronutrient levels emerges as a new strategy or agronomic practice to reduce water consumption and improve drought resistance in both horticultural crops and other species of agronomic interest.

## 5 Chapter 4—Soil Management and Conservation Practices

**FIGURE 4:**
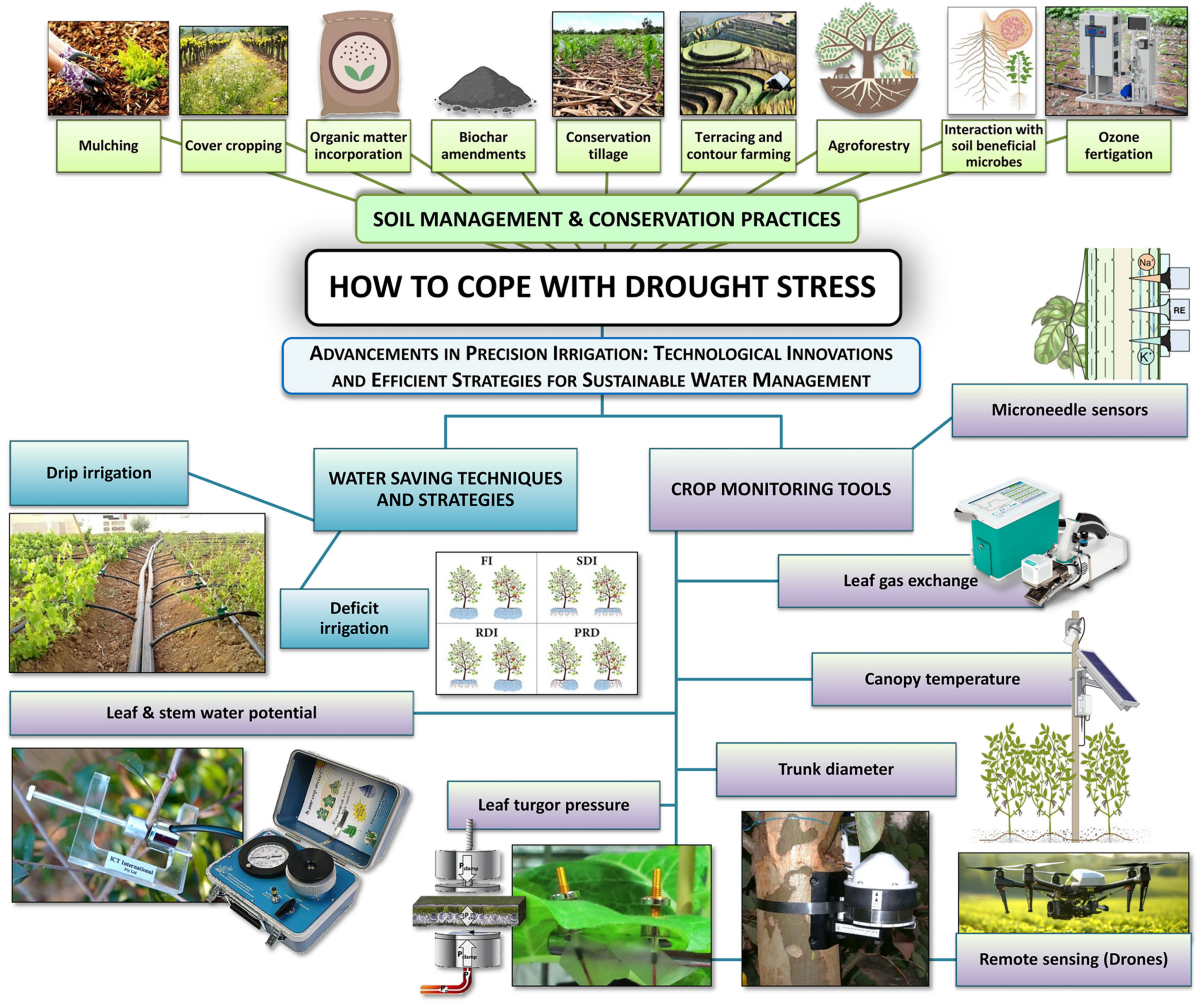
Schematic representation of the main aspects of Chapter 4 (Soil management and conservation practices) and Chapter 5 (Advancements in precision irrigation: Technological innovations and efficient strategies for sustainable water management). Some of the equipment shown are following: Deficit irrigation techniques (Laita et al. 2024); dendrometer DRL26D; HIDRO VT GZO40‐EPS professional ozone (O_3_) generator (Zonosistem, El Puerto de Santa María, Spain, https://www.zonosistem.com/); high resolution band dendrometers for trees and plants (DB‐60); ICT leaf and canopy temperature sensor (SKU‐IOT); leaf gas exchange and photosystem II fluorescence analyser (Li‐6800 portable photosynthesis system); Microneedle sensors (Wang, Molinero‐Fernández, et al. 2024); PSY1 psychrometer (stem & leaf) for plant Ψ_w_ (Ψ_w,leaf_ and Ψ_w,stem_); real‐time measurement of leaf turgor using the non‐invasive magnetic leaf patch‐clamp pressure probes (Zimmermann et al. 2008); Schölander chamber for plant Ψ_w_ (Model 1000). *Source:* Parts of the images were fully provided with permission from J.D. Franco‐Navarro's thesis (Franco‐Navarro 2022). Most elements of this scheme were created with BioRender.com (CC‐BY 4.0 license).

Soil management and conservation practices are essential components of sustainable agriculture, playing a critical role in enhancing soil health, productivity, and ecosystem resilience. As the foundation of agricultural systems, soil quality directly influences crop yields and environmental sustainability (Blanco and Lal 2023). Effective soil management involves techniques that promote soil structure, fertility, and biodiversity while minimizing degradation caused by erosion, compaction, and nutrient depletion (Raj et al. 2023). Conservation practices, such as cover cropping (Won et al. 2024), reduced tillage (Bezboruah et al. 2024; Sadiq et al. 2021), mulching (Prem et al. 2020), and agroforestry (Fahad et al. 2022), not only help preserve soil integrity but also enhance water retention, promote carbon sequestration, and reduce the impact of climate change and drought stress. Additionally, these practices minimize water loss through evaporation and maintain soil structure, contributing to a more sustainable and resilient agricultural landscape that supports both food production and environmental conservation (Dubey et al. 2024; Grover et al. 2024; Tahat et al. 2020; White et al. 2012; Figure 4).

Soil health plays a pivotal role in enhancing drought resilience and other stresses, serving as a natural buffer against water scarcity (Dewi et al. 2023). Healthy soils with well‐structured aggregates and high organic matter content improve water infiltration and retention, ensuring that plants have access to moisture even during dry periods. Soil organic matter acts like a sponge, holding water and releasing it gradually, which reduces the risk of crop stress during droughts (Mohanty et al. 2024). Additionally, healthy soils support diverse microbial communities that enhance root growth and nutrient uptake, enabling plants to access deeper soil layers for water and nutrients (Singh et al. 2024).

### 5.1 Mulching

Mulch, derived from natural materials such as straw, increases soil organic matter, which improves the soil's water‐holding capacity (Verma and Pradhan 2024). Mulching is a common and effective practice used to conserve soil moisture and improve drought tolerance or resistance in wheat (Zhang et al. 2023), maize (Zambrano et al. 2024), potato (Song et al., 2024), and bean (Uwanyirigira et al. 2023). By covering the soil surface with a layer of organic or synthetic materials, such as straw, wood chips, plastic, or biodegradable films, mulch acts as a protective barrier that reduces soil moisture evaporation by shielding the soil from direct sunlight and wind exposure while building soil organic matter (Prem et al. 2020). Additionally, mulch helps regulate the soil temperature, reduces N$O_{3}^{-}$ leaching, suppresses weed growth, and improves soil structure, thereby promoting water infiltration and retention in maize plants (Dai et al. 2021).

### 5.2 Cover Cropping

Cover crops are plants grown primarily for the benefit of the soil rather than for crop yield (Won et al. 2024). Cover cropping is a fundamental agricultural practice that involves planting non‐harvested crops or specific plant species, known as cover crops, during periods when the main cash crop is not growing. These cover crops play a crucial role in managing various aspects of soil health and agricultural sustainability. For instance, cover crops are essential for preventing soil erosion, enhancing soil fertility, improving soil quality, regulating water availability, controlling weeds, pests, and diseases, promoting biodiversity, and supporting wildlife in agroecosystems (Van Eerd et al. 2023). They contribute to increasing microbial activity in the soil, positively impacting N availability, uptake in target crops, and overall crop yields. Cover crops are particularly effective in reducing soil loss by enhancing soil structure, increasing infiltration, protecting the soil surface, and reducing water movement velocity over the soil surface (Van Eerd et al. 2023; Won et al. 2024). The dense root networks of cover crops help anchor the soil, increase soil porosity, and create suitable habitats for soil macrofauna, thereby enriching the soil for future agricultural productivity. Common cover crop species include *Secale cereale* (rye), *Vicia villosa* (hairy vetch), *Trifolium pratense* (red clover), *Sorghum bicolor* (sorghum‐sudangrass), and various species in the Brassicaceae family (Mennan et al. 2020). Research studies have shown that cover crops can significantly contribute to weed control, with rye cover crop residues providing substantial control of early‐season broadleaf weeds when used as mulch during the production of soybean (Kumari et al. 2023). Additionally, strategic planting of cover crops in conjunction with cash crops can lead to a remarkable reduction in weed growth, providing farmers with valuable insights into optimizing their cropping rotations for enhanced agricultural sustainability.

### 5.3 Organic Matter Incorporation

Incorporating organic matter into soil, whether through compost, manure, crop residues, or green manure, offers a multitude of advantages for soil health and productivity (Singh, Sharma, et al. 2023; Singh, Bijay‐Singh, et al. 2023; Singh et al. 2022; Singh and Benbi 2023). Organic matter serves as a vital component in enhancing soil structure, water retention, and nutrient availability. Acting as a sponge, organic matter effectively retains water within soil pores, thereby reducing rapid evaporation and ensuring a more consistent moisture level in the soil. This water‐holding capacity not only supports plant growth, but also aids in mitigating the effects of drought stress (Nieder et al. 2024).

Furthermore, the presence of organic matter in soil fosters a conducive environment for microbial activity and decomposition processes (Singh et al. 2022). These activities play a crucial role in breaking down organic materials, releasing essential nutrients, and forming stable soil aggregates (Li, Wang, et al. 2023; Li, Piao, et al. 2023). The formation of these aggregates enhances soil structure, promoting better aeration, root penetration, and overall soil health (Nieder et al. 2024). Additionally, the increased microbial activity facilitated by organic matter contributes to nutrient cycling, making vital nutrients more readily available to plants for uptake (Siles et al. 2024).

### 5.4 Biochar Amendments

Biochar is a soil amendment based in C‐rich solid residue produced through thermochemical decomposition of waste biomass, typically under oxygen‐limited environments (pyrolysis) and has been a trending technique in sustainable agriculture in the last decade (Paneque et al. 2016). The utilization of this carbonaceous residue is coherent with modern green agriculture, as it has been found to control soil pollution, for example, heavy metals, pesticides, among others (Bolan et al. 2024). It also balances the soil organic and inorganic matter, improves biological properties (microbial activity) and soil physical properties such as pH, cation exchange capacity, pore size distribution, bulk density, soil structure, soil organic carbon, and soil water holding capacity (Atkinson et al. 2010; Godlewska et al. 2021; Omondi et al. 2016). Biochar reduces the loss of nutrients due to leaching and increases the bioavailability of soil nutrients (Chen et al. 2021; Igalavithana et al. 2017). Soil fertility and productivity are enhanced while improving the quality and structure of degraded soils when biochar is used (Khan et al. 2024; Sharma et al. 2024; van Zwieten et al. 2010).

The effectiveness of biochar for promoting plant production and crop yield has been demonstrated in species like rice (Medyńska‐Juraszek et al. 2021), wheat (Amer 2017; Olmo et al. 2014), maize (Medyńska‐Juraszek et al. 2021; Omondi et al. 2016; Yamato et al. 2006), the common bean (Raboin et al. 2016), soybean (Lee et al. 2000), sweet potato (Indawan et al. 2018), potato (Mollick et al. 2020), onion (Aneseyee and Wolde 2021), carrot (Carpenter and Nair 2016), sunflower (Paneque et al. 2016), and tobacco (Tepecik et al. 2024).

### 5.5 Conservation Tillage

Conservation tillage practices, such as no‐till, minimum tillage, and reduced tillage, have garnered significant attention in recent years due to their numerous benefits for soil health and sustainability (Bezboruah et al. 2024). These practices aim to minimize soil disturbance and maintain crop residues on the soil surface, which in turn reduces soil erosion, conserves soil moisture, and mitigates evaporation (Sadiq et al. 2021). By leaving crop residues intact, conservation tillage creates a protective layer on the soil surface, preventing the direct impact of raindrops and reducing the risk of soil particles being dislodged and carried away by water or wind. Moreover, conservation tillage practices have been shown to enhance soil organic matter content, a crucial component for maintaining soil health (Bezboruah et al. 2024). As crop residues decompose on the soil surface, they contribute to the accumulation of organic matter, which improves soil structure and water‐holding capacity. The enhanced soil structure, characterized by stable aggregates, allows for better infiltration of water, reducing surface runoff and increasing the amount of water available for plant uptake. In addition to improving the soil's physical properties, conservation tillage practices also foster beneficial soil microbial communities (Khan, Awan, et al. 2023; Khan, Liu, et al. 2023; Khan, Liu, et al. 2023). These microorganisms play a vital role in nutrient cycling, decomposition of organic matter, and the formation of stable soil aggregates (Lv et al. 2023). By maintaining a diverse and active microbial community, conservation tillage contributes to the overall resilience and productivity of the soil ecosystem.

### 5.6 Terracing and Contour Farming

Terracing and contour farming are soil conservation techniques that play a crucial role in mitigating soil erosion, enhancing water retention, and promoting sustainable land management practices. These techniques involve shaping the land into terraces or contour lines, creating level surfaces or ridges along the natural contours of the land. By doing so, terracing and contour farming effectively reduce water runoff and soil erosion, allowing water to infiltrate into the soil and replenish groundwater resources (Thompson and Sudduth 2018). Particularly effective on sloping terrain, terracing and contour farming help to conserve soil moisture and prevent soil degradation by controlling the flow of water across the landscape (Kurdekar et al. 2023). The terraces and contour lines act as barriers, slowing down the movement of water and preventing it from carrying away valuable topsoil. This not only reduces erosion but also promotes the retention of soil nutrients and organic matter, essential for sustaining healthy and productive soils (Kumar et al. 2023). Furthermore, these soil conservation techniques contribute to the overall health of the ecosystem by maintaining soil structure, fostering plant growth, and supporting biodiversity. By creating a more stable environment for vegetation to thrive, terracing and contour farming help to prevent landslides, improve water quality, and enhance the resilience of the landscape against environmental challenges (Sharma et al. 2023).

### 5.7 Agroforestry Systems

Agroforestry systems represent a sustainable and integrated approach to land management that combines trees or shrubs with agricultural crops, offering a range of benefits for soil health, water retention, and biodiversity conservation. By incorporating trees into agricultural landscapes, agroforestry systems contribute to enhanced soil fertility, improved water retention, and increased biodiversity (Fahad et al. 2022). The presence of trees in agroforestry systems plays a significant role in improving soil structure and fertility. Tree roots help to break up compacted soil, allowing for better water infiltration and root penetration (Zaib et al. 2023). Additionally, the leaf litter and organic matter produced by trees contribute to the soil's nutrient content and overall health. Trees also play a crucial role in reducing soil moisture loss through transpiration and shading, creating a more favorable microclimate for plant growth and soil conservation in arid and extreme drought stress environments (Zhao, Gao, An, et al. 2023; Zhao, Duan, et al. 2023; Zhao et al. 2022). Agroforestry practices such as alley cropping, windbreaks, and silvopasture offer a multitude of benefits beyond soil improvement. Alley cropping involves planting rows of trees or shrubs between crop rows, providing shade, wind protection, and organic matter inputs (Quinkenstein et al. 2009). Windbreaks help to reduce soil erosion and protect crops from wind damage (Vacek et al. 2018), while silvopasture integrates trees with livestock grazing, enhancing climate change mitigation and providing additional income streams for farmers (Greene et al. 2023).

### 5.8 Interactions With Soil Beneficial Microbes

The rhizosphere contains more than 30,000 different species of microorganisms (plant growth‐promoting rhizobacteria, PGPRs; bio‐control Agents, BCAs; saprophytes, etc.) that represent a new paradigm for agriculture. The use of microorganisms in agriculture has been established as a sustainable and effective opportunity to improve plant development, productivity, and quality of crops (Elnahal et al. 2022; Mącik et al. 2020). Under drought conditions, plant‐associated microorganisms play important roles in different biological processes in the soil that affect plant development and defense mechanisms. A large number of bacteria and fungi can provide adaptive advantages to plants against drought through physiological and biochemical mechanisms. Their functions differ from biofertilizers, biocontrollers, phytostimulators, or bioprotectors to the combination of several of them (Abdelaal et al. 2021; Kumar et al. 2019). Hence, their use is of great importance in the search for new alternatives to mitigate the consequences caused by water scarcity. However, the effect of using these microbial agents is conditioned by different factors, such as the type of soil or substrate, culture medium, and species of plant and microorganisms used, as well as the duration and level of stress to which they are subjected (Mardukhi et al. 2011; Ruiz‐Lozano et al. 1995). The development of sustainable strategies, such as the use of specific microorganisms, that is, PGPRs and arbuscular mycorrhizal fungi (AMF), can increase abiotic stress tolerance, boost plant growth, improve nutrient uptake, and reduce reliance on agrochemicals to combat the effects of drought (Egamberdieva 2012; Hnini et al. 2024; Kour and Yadav 2022).

Harnessing the potential of these microbes as sustainable agricultural practices can increase crop productivity, improve soil health, and promote the long‐term sustainability of agroecosystems in water‐constrained environments. It is now accepted that the contribution of microbial symbiosis to plant drought resistance is the result of the accumulation of physical, nutritional, physiological, and cellular effects. Beneficial microbes, including mycorrhizal fungi and rhizobacteria, play a crucial role in enhancing drought resilience in plants through various mechanisms, which often work in combination rather than in isolation (Banerjee and van der Heijden 2023; Zayed et al. 2023).

#### 5.8.1 Improved Nutrient Uptake

Microorganisms are key contributors to nutrient use efficiency in plants, particularly in environments typically characterized by nutrient limitations (Smith and Read 2008). Mycorrhizal fungi form symbiotic associations with plant roots, extending their hyphal networks into the soil to increase the surface area for nutrient uptake. In doing so, they enhance water and nutrient absorption and improve plant resistance to environmental stress (Khaliq et al. 2022). This improves the plant's ability to acquire essential nutrients, such as phosphorus and N, which are vital for maintaining physiological functions under drought conditions. In return, the fungus takes nutrients in the form of organic carbon from the host plant to further its growth and development. Many authors have reported that AMF are capable of increasing the assimilation of nutrients such as P, K, and N under different abiotic stress conditions that depend on the distribution of photoassimilates among associated organisms (Ahanger et al. 2014; Aroca et al. 2013; Gómez‐Bellot, Nortes, et al. 2015; Gómez‐Bellot, Ortuño, et al. 2015). Some authors confirmed that AMF improved the capacity of plants to absorb P and N and organic carbon from the soil in horticultural plants under deficit irrigation, which helped to improve their photosynthetic capacity and WUE (Badr et al. 2020; Liu et al. 2018; Sánchez‐Blanco et al. 2004). In arid conditions, AMF are also able to improve phosphorus uptake from the soil (Caravaca et al. 2005). AMF‐associated rice showed a reduction in the transcript levels of two transporter genes (*PT2* and *PT6*) involved in direct P‐uptake (Jeong et al. 2015). This may explain the significantly higher P‐uptake via the AMF‐mediated pathway rather than direct root uptake. Certain rhizobacteria, such as N‐fixing bacteria (e.g., rhizobia and diazotrophs) have the ability to convert atmospheric N (N_2_) to ammonia (NH_3_) through N fixation. This process enriches the soil with available N, which is essential for plant growth and metabolism. Plants associated with N‐fixing bacteria can access a sustainable source of N even in N‐deficient soils, thus alleviating nutritional stress (Zayed et al. 2023).

#### 5.8.2 Enhanced Water Absorption

Mycorrhizal fungi facilitate the uptake of water by extending their hyphae deep into the soil, helping plants access moisture during periods of drought stress (Augé 2004). Symbiosis with AMF may change the water movement through the host plants, affecting plant hydration and physiology. When AMF alter the water relations of the plant and the soil under stress conditions, *g*_s_, photosynthetic rate, and transpiration in plants are modified (Liese et al. 2017; Morte et al. 2010; Zou et al. 2014). A number of studies have demonstrated that during soil drying, mycorrhizal plants often maintain higher gas exchange rates than non‐mycorrhizal plants (Porcel and Ruiz‐Lozano 2004). Traditionally, it has been suggested that the water status of mycorrhizal plants is related to nutritional aspects associated with phosphorus (Augé et al. 2001). In contrast, other studies have shown that water relations and gas exchange in mycorrhizal plants may be altered independently of phosphorus nutrition (Morte et al. 2001). Similarly, other authors have discussed the importance of the effect of mycorrhizae on gas exchange and Ψ_w,leaf_ in several species (Dell'Amico et al. 2002; Gómez‐Bellot, Nortes, et al. 2015; Gómez‐Bellot, Ortuño, et al. 2015; Vicente‐Sánchez et al. 2014). Mycorrhization has also been observed to contribute to improved water uptake by roots (Allen 1982), through increased soil water efficiency (Gómez‐Bellot, Nortes, et al. 2015; Gómez‐Bellot, Ortuño, et al. 2015). Under drought conditions, rhizobacteria also use water strategies that increase the Ψ_w_ of the plant, the water content in leaves, the apoplastic fraction of water, or hydraulic conductivity (Bittencourt et al. 2023; Creus et al. 2004; Rincón et al. 2008). The mechanisms by which these effects take place are still not well defined, although several authors have reported that the combination of enzymatic or hormonal mechanisms, such as ABA, and other nutritional mechanisms, is involved in the improvement of plant water relations under drought conditions (Ngumbi and Kloepper 2016).

#### 5.8.3 Osmotic Adjustment to Overcome Drought and Stress Tolerance

Beneficial microbes produce osmolytes and other compatible solutes that help plants maintain cellular Ψ_P_ and osmotic balance under water stress conditions (Abdelaal et al. 2021). To achieve this, plants decrease their Ψ_w_ to maintain a favorable gradient for the flow of water from the soil to the roots through osmotic adjustments. The Ψ_π_ in the leaf decreases through the active accumulation of organic ions or solutes (Kubikova et al. 2001). Proline is one of the best‐known osmoprotectants, and its synthesis mitigates the adverse effects of drought‐induced water stress on plant cells. Proline, together with glycine betaine and trehalose, increases the thermotolerance of enzymes, inhibits thermal denaturation of proteins, and helps maintain membrane integrity (Bérard et al. 2015; Schimel et al. 2007). The production of solutes, spores, and exopolysaccharides promoted by bacteria helps to protect cellular structures and organelles (Bérard et al. 2015). It has also been observed that plant‐mycorrhizae associations modify the reserves of free amino acids and sugars in the roots (Ruiz‐Lozano et al. 2012).

#### 5.8.4 Induction of Systemic Resistance of Plant's Immune System

Rhizobacteria and mycorrhizal fungi can stimulate the plant's innate immune system, triggering the production of defence‐related compounds and antioxidant enzymes. This systemic resistance response primes plants to withstand drought stress more effectively by activating stress‐responsive pathways and enhancing their capacity to scavenge ROS. It has been proven that some bacteria such as *Bacillus subtilis* promote the accumulation of osmoprotectants such as amino acids and sugars and accelerate the production of starch and antioxidants (El‐Beltagi et al. 2023; Gagné‐Bourque et al. 2016), stimulate drought‐responsive genes, and exert an influence on DNA methylation (Shalaby et al. 2023). AMF have been observed to be capable of improving plant resistance to water stress by regulating enzymatic and non‐enzymatic antioxidant defence systems to scavenge ROS, via metabolic changes and the induction of genes for scavenging ROS (*Cu/Zn‐SOD*, *GRX1*, *MT1*, *PDX1*, *Rboh*, *SOD1*; Zou et al. 2021). The effects of AMF on the antioxidant system of plants were shown to contribute to the maintenance of redox homeostasis and improve the protection of the metabolic pathways, including nitrogen assimilation and photosynthesis. This involves the up‐regulation of the AsA–GSH cycle, enhancement of nitrate reductase (NR) activity and N uptake favouring the synthesis of stress‐protective amino acids like proline, and protection of Rubisco activity via glycine betaine accumulation, ultimately supporting photosynthetic efficiency under stress (Begum, Qin, et al. 2019; Begum, Ahanger, et al. 2019).

#### 5.8.5 Production of PGPRs

Beneficial microbes produce phytohormones, such as auxins, cytokinins, and gibberellins, or inhibit others, such as ethylene, which regulate plant growth and development, including the stimulation of root development and the water balance (Curá et al. 2017; Paul and Lade 2014). PGPRs such as *Bacillus amyloliquifaciens* typically reside in the soil and establish symbiotic relationships with many plants, producing gibberellins, cytokinins, auxins, and polyamines, directly influencing root and root hair growth (Xie et al. 2014). This helps in the uptake of water and nutrients under drought conditions (García‐Fraile et al. 2015; Kumari et al. 2018). Proteins such as polyamines are also produced under drought conditions, particularly during osmotic stress, as observed in maize inoculated with *Azospirillum* sp. and *Herbaspirillum* sp., where their accumulation was associated with improved root growth and mitigation of hydric deficit (Curá et al. 2017). Indoleacetic acid is also commonly produced by 80% of N_2_‐fixing bacteria, which have been observed to enhance the activity of the polyphenol oxidase enzyme and increase plant Ca^2+^ content and total phenols (Ahmad et al. 2008). All these mechanisms protect the plant against pathogens and help to eliminate ROS (Chowdhury 2003). Several studies have also confirmed that inoculation with mycorrhizal fungi results in decreasing endogenous ABA and SA (Aroca et al. 2013; Ren et al. 2019; Torres et al. 2018). Such changes in phytohormones by AMF would provide clues about the enhanced drought tolerance in the host plant (Wu and Zou 2017). In addition, other studies reported that ABA content increased in plants inoculated with mycorrhizae under water stress conditions, as observed in *Ephedra foliate* and *Olea europaea* cultivars. In *E. foliate*, AMF‐inoculated plants maintained higher ABA levels during drought, which contributed to enhanced stress tolerance. Similarly, in olive trees, increased ABA accumulation in inoculated “Zarrazi” plants under moderate drought and recovery phases correlated with improved resilience. These findings suggest that AMF modulate ABA dynamics in a species‐ and cultivar‐dependent manner, influencing drought responses beyond mere stomatal regulation (Al‐Arjani et al. 2020; Ouledali et al. 2019).

### 5.9 Formation of Biofilms and Soil Aggregation

Soil microorganisms contribute to the formation of biofilms and soil aggregates through the deposition of extracellular polysaccharides and the formation of degraded humic materials, which stabilize soil structure and prevent erosion. This soil aggregation enhances water infiltration and retention, reducing the risk of water runoffs and soil moisture loss during drought periods (Blankinship et al. 2016). In addition, both fungi and bacterial inocula increase the availability of nutrients in the soil solution through the decomposition of organic matter, the fixation of N, and the mobilization of P, K, and Fe (Carrasco‐Chaico 2021). AMF have the ability to improve soil structure due to their chemical and biological actions and their exudates, which increase water infiltration and retention capacity in the rhizosphere (Jastrow and Miller 1991; Oades and Waters 1991; Rillig and Mummey 2006). In addition, AMF produce a glycoprotein that helps in the creation of soil aggregates and protects hyphae from the loss of water and nutrients. Several authors have reported that the content of easily extractable glomalin in soil increased when plants subjected to abiotic stresses were inoculated with AMF, improving soil stability (Gómez‐Bellot, Nortes, et al. 2015; Gómez‐Bellot, Ortuño, et al. 2015; Wang et al. 2023; Zou et al. 2014). On the other hand, during drought conditions, PGPR may act as biofertilizers by facilitating the uptake of specific nutrients through various mechanisms. These encompass mineral solubilisation, fixation of N, absorption of K^+^ or phosphate (P$O_{4}^{3-}$), iron sequestration, and production of siderophores (Chieb and Gachomo 2023; Rizvi et al. 2022). In any case, numerous authors have reported that the co‐inoculation of microbes, such as certain bacteria and fungi, could be more beneficial than the application of a single microbe to improve the bioavailability of nutrients in the soil (Bona et al. 2018; Khalid et al. 2017; Todeschini et al. 2018) even in drought conditions (Musyoka et al. 2020).

#### 5.9.1 Microorganisms Improve Plant's Physiology Under Drought

Microorganisms in general can improve plant quality parameters through their effects on the physiological and morphological response of plants, allowing them to better overcome stress during recovery (Ma et al. 2009). The application of some Plant Growth‐Promoting Bacteria (PGPB) can improve the quality of plants both under normal conditions and under abiotic stresses, increasing the number of flowers and increasing plant growth in ornamental species (Hoda and Mona 2014; Leoni et al. 2019). Nordstedt and Jones (2020) demonstrated that the application of certain bacteria improved chlorophyll fluorescence parameters and electrolyte loss, and increased plant size and the number of flowers in ornamental species after a period of water stress. Associations with AMF can also increase the production of secondary metabolites (Noceto et al. 2021; Sharma et al. 2017), which may correspond to increases in foliage color. Some studies have shown that AMF improve fruit and flower production, inducing early flowering and increasing flowering time (Burkle and Zabinski 2023; Sangwan et al. 2023; Sangwan and Prasanna 2022). However, these effects can also be detrimental to vegetative growth (Gaur and Adholeya 2005; Linderman and Davis 2004).

### 5.10 Ozone (O_3_ ) Fertigation

Ozone (O_3_) is an oxidizing agent and also a powerful disinfectant (Risoli and Lauria 2022). It is used in the food industry (İbanoğlu 2023), cleaning services (Saqib et al. 2024), healthcare facilities (Sousa et al. 2011), and the recycling of wastewater (İbanoğlu 2023; Saqib et al. 2024).

Agriculture is another promising field where O_3_ has demonstrated significant benefits over the past decade. The application of ozonated water at low concentrations (< 10 mg L^−1^), whether through foliar spraying, soil irrigation, seed treatment, or incorporation into hydroponic nutrient solutions, has proven to boost plant performance while leaving no chemical residues on crops. These treatments have been associated with increased plant biomass, chlorophyll content, vitamin C levels, secondary metabolite production, and antioxidant activity, while also contributing to the reduction of pests and diseases (Risoli and Lauria 2022).

O_3_ fertigation, the process of injecting ozonated water into drip irrigation systems, has shown promising results in enhancing irrigation efficiency and promoting plant health (Risoli and Lauria 2022). One key benefit of ozonated water is its ability to disinfect irrigation water by eliminating harmful pathogens, bacteria, and viruses (Li and Wang 2003; Yamamoto et al. 1990). This ensures cleaner water delivery to crops, reducing the risk of plant diseases. O_3_ also helps in removing and preventing biofilm formation in drip irrigation tubes, which can obstruct water flow and reduce system efficiency (Graham et al. 2011). By keeping the tubes clean, it improves water distribution and prevents clogging issues.

Ozonated water also positively impacts soil health by increasing soil fertility and improving its physicochemical and biological properties. O_3_ oxidizes organic matter and converts it into simpler, plant‐available nutrients, promoting nutrient uptake (Mathew et al. 2024). Additionally, O_3_ enhances soil aeration and microbial activity, contributing to healthier root environments and boosting overall plant growth (Graham et al. 2011).

Under both water‐limited and optimal irrigation conditions, O_3_ fertigation has been shown to improve overall water balance and WUE in several crops, including corn (Monteiro et al. 2021), cucumber (Najarian et al. 2018; Najarian et al. 2015), lettuce (Monteiro et al. 2021), pepper (Colunje et al. 2021; Martínez‐Sánchez and Aguayo 2019), and tomato (Guo et al. 2019; Prigigallo et al. 2019; Veronico et al. 2017), thereby enhancing drought resistance. These benefits are associated with improved root development, increased uptake of essential nutrients (N, P, and K), and higher chlorophyll content, all contributing to greater plant growth, vigor, and fruit biomass. In addition, O_3_‐induced enhancement of antioxidant capacity improves the scavenging of ROS, thereby mitigating the oxidative stress typically associated with drought.

Overall, O_3_ fertigation offers multiple benefits through a process of hormesis, in which moderate and usually intermittent stress stimulates beneficial effects. These include increased antioxidant enzyme activity, enhanced physiological processes, healthier crop growth, and improved resilience to environmental stresses such as drought, through better water retention and more efficient nutrient uptake via the roots (Agathokleous et al. 2019).

## 6 Chapter 5—Advancements in Precision Irrigation: Technological Innovations and Efficient Strategies for Sustainable Water Management

Global warming exacerbates the challenges of water scarcity, particularly in arid and Mediterranean regions where water resources are already limited (Chen et al. 2022). In this context, precision irrigation represents a comprehensive and advanced approach that incorporates efficient irrigation techniques and strategies, advanced technologies and tools, and a coordinated strategy (Fernández 2017). This approach not only conserves water resources but also promotes environmental sustainability and ensures high crop productivity. Precision irrigation becomes essential for sustainable water management in light of future water scarcity prospects (Figure 4).

Precision irrigation has emerged as a critical tool in sustainable agriculture. First, it mitigates the adverse effects of drought by delivering water precisely where and when it is needed, thereby maintaining crop productivity under water‐limited conditions, thus ensuring food security. Second, it significantly enhances WUE by optimizing irrigation schedules and minimizing losses, a key advantage in regions facing water scarcity. Finally, precision irrigation contributes to long‐term water conservation and strengthens agricultural resilience to climate variability by reducing unnecessary water wastage and preserving vital water resources to build resilience against changing climatic conditions (Ahmed et al. 2023; Bwambale et al. 2022; Lakhiar et al. 2024).

This section delves into the pillars of precision irrigation, focusing on the technological tools used for monitoring crop water needs and the efficient irrigation techniques that enhance WUE and water productivity. Precision irrigation can help to improve water resource management by improving WUE and maintaining crop yield.

### 6.1 Water‐Saving Irrigation Techniques

#### 6.1.1 Drip Irrigation Advancements

Drip irrigation systems represent a significant advancement in irrigation, particularly for tree crops and other plants that do not cover the entire ground surface, as water evaporation from the soil surface is reduced, increasing WUE (Wang et al. 2021). One of the primary distinctions between localized drip irrigation and full coverage systems is the volume of wetted soil. While full coverage systems are traditional systems that aim to wet nearly 100% of the soil surface, drip irrigation significantly reduces this percentage to minimize water loss through evaporation from the soil surface. Drip irrigation delivers water directly to the root zone of plants, ensuring efficient water use. As compared to other irrigation methods, drip irrigation systems allow the application of lower volumes of water more frequently and efficiently. When properly designed, these systems can deliver water and nutrients directly to the plant's root zone, while minimizing evaporation and deep percolation, being more efficient than traditional irrigation systems and allowing for water savings.

#### 6.1.2 Agronomic Design for an Efficient Irrigation

Optimizing water distribution and aligning irrigation with crop water requirements ensures uniform water application, matching the specific needs of each crop type and growth stage. In drip irrigation, the number of emitters per plant determines the number and dimensions of the wet bulb, thereby determining the volume of the wetted soil. The root system, in arid and semiarid areas, tends to be confined to this wetted area, where conditions are optimal, as both water and nutrients are available (García‐Tejera et al. 2018). The volume of wetted soil significantly affects a crop's response, even when the water supply is sufficient to theoretically meet the crop's water needs. This highlights the importance of agronomic design in improving the physiological response of crops. Therefore, the design of the drip irrigation system is highly relevant. Especially in arid and semiarid areas, the effect of a larger portion of wetted soil resulting from an increased number of emitters per plant shows a positive effect on the crops´ water status, mainly when evapotranspirative demand is high. In this sense, increasing the volume wetted by irrigation in almonds led to an increase in transpiration and growth (Espadafor et al. 2018), suggesting that the trees' potential growth and productivity may be limited as a consequence of a small volume of wetted soil. In lemons, it has been observed that increasing from two drip lines in traditional irrigation, to three lines expanded the wetted soil surface. In this way, a better distribution of water in the soil profile was promoted, locating it to the area with the highest concentration of roots. Therefore, the physiological response was improved, as well as the WUE (Robles et al. 2023).

#### 6.1.3 Sub‐Surface Drip Irrigation (SDI)

Sub‐surface drip irrigation (SDI) is a variation of traditional drip irrigation, where driplines are buried beneath the soil rather than placed on the surface. This irrigation system delivers water directly to the root zone while keeping the soil surface dry, thereby minimizing water loss from evaporation and preventing weed growth. SDI has been proposed as a promising strategy for sustainable water management in semiarid regions. SDI has demonstrated significant water savings, as compared to surface drip irrigation (SI), without reducing yield and increasing WUE. In the case of citrus trees in semiarid climate conditions, the adoption of SDI achieved water savings between 20% and 25%, without affecting yields (Martínez‐Gimeno et al. 2018; Robles et al. 2016).

### 6.2 Water‐Saving Irrigation Strategies

#### 6.2.1 Deficit Irrigation (DI) Strategies

Deficit irrigation (DI) strategies involve applying water at levels below the evapotranspiration requirements of the crop (Fereres et al. 2006).

#### 6.2.2 Sustained Deficit Irrigation (SDI)

Sustained deficit irrigation (SDI) consists of applying a certain WD during the entire growing season. DI aims to deliver a uniform and below‐optimal amount of water irrigation. By applying water below the maximum crop evapotranspiration (ET_c_), SDI helps improve WUE while maintaining adequate crop yield and increasing water productivity. This irrigation strategy has been successfully applied to olives, almonds (Egea et al. 2013), and citrus trees (Garcia Tejero et al. 2011), increasing water productivity.

#### 6.2.3 Regulated Deficit Irrigation (RDI)

Regulated deficit irrigation (RDI) involves reducing irrigation below the plant's water needs during the less critical periods of the crop, while adequately meeting water requirements during the rest of the phenological cycle, so as to not compromise either the yield or the quality of the fruits. By regulating the timing, duration, and severity of the imposed water stress, RDI helps to improve WUE and/or fruit quality. RDI was first proposed as an irrigation method to control vegetative vigor in peaches (Chalmers et al. 1981). RDI experiments with many tree crops in the Mediterranean regions, such as olive (Martínez‐Gimeno et al. 2022), almond (Girona et al. 2005), or citrus trees (Pérez‐Pérez et al. 2009), confirmed the feasibility of RDI to improve water productivity. However, RDI requires detailed knowledge of crop phenology and water needs (Gómez Álvarez et al. 2009).

#### 6.2.4 Partial Root Drying (PRD)

Partial root drying (PRD) is a deficit irrigation technique designed to improve WUE by applying water to only a portion of the plant's root zone. With this technique, one part is irrigated while the other part is left to dry. PRD is based on root‐to‐shoot signalling, involving ABA, which controls the plant's response to soil drying (Dodd 2005). The chemical signals produced in the drying roots decrease stomatal conductance and limit vegetative vigor, while the well‐hydrated roots help to maintain a favorable water status (Dodd et al. 2009). This approach reduces overall water consumption while maintaining crop productivity, making it an effective strategy for sustainable water management. This technique was successfully applied in citrus trees (Saitta et al. 2021).

#### 6.2.5 Alternate Wetting and Drying Irrigation (AWD)

Alternate wetting and drying irrigation (AWD) is an irrigation technique very used in rice that repeatedly dries and re‐floods fields (Acosta‐Motos et al. 2020). In this sense, alternate wetting and drying irrigation can enhance rice WUE without significantly affecting crop yield (Carrijo et al. 2017).

### 6.3 Crop Monitoring Tools for Assessing Plant Water Status

Plant monitoring is used not only for evaluating water stress and optimizing irrigation scheduling, but also for detecting early signs of disease, nutrient deficiencies, and other environmental stresses, thereby supporting timely and targeted crop management decisions (Ajith et al. 2025; Velazquez‐Chavez et al. 2024). This approach relies on using the plant as a biosensor, integrating soil and atmospheric water status with the plant's physiological response to available water (Fernández 2017). The use of these tools to directly or indirectly measure crop water status involves a significant advancement in precision irrigation. This approach helps prevent water stress, which can negatively impact fruit quality and yield, as well as overwatering. The determination of crop water status plays a key role in supporting the adoption of sustainable irrigation practices and the implementation of precision irrigation. Plant water status can be evaluated by direct measurements of leaf and stem water status or indirectly by measuring leaf gas exchange, trunk diameters, sap flow, leaf turgor, or canopy temperature. These indicators are useful for managing irrigation efficiently, to achieve substantial water savings without compromising productivity.

Artificial intelligence, particularly machine learning and deep learning, is revolutionizing agricultural practices by enabling data‐driven, precise, and sustainable solutions across domains such as crop yield prediction, precision irrigation, soil fertility mapping, pest and disease forecasting, and foodgrain quality assessment. In recent years, monitoring crop water status and stress has increasingly focused on plant‐based sensors equipped with data transmission systems, allowing data to be recorded automatically and continuously. These systems capture measurements related to sap flow, trunk diameter, and leaf Ψ_P_, among other variables, providing a robust platform for AI‐assisted analysis and decision‐making in irrigation scheduling and stress detection (Ajith et al. 2025).

These methods have the advantage of running continuously and automatically, and they can be implemented with data transmission systems for easy and remote access to the recorded data (Ahmad et al. 2021; Ihuoma and Madramootoo 2017; Simbeye et al. 2023; Wang et al. 2010). Moreover, future trends in instruments for both soil and plant monitoring are detailed below.

### 6.4 Leaf and Stem Water Potential (Ψ_w,leaf_ and Ψ_w,stem_)

Ψ_w_ is related to plant water status and has been widely used as a water stress indicator for irrigation management. Ψ_w_ is measured with a pressure chamber that measures the hydrostatic pressure contained in the plant xylem. This is the most widely used methodology to measure Ψ_w_. In the pressure chamber, a positive pressure is applied to an excised plant section inside a chamber until the liquid content of the sample is forced out. At this point, the applied pressure is Ψ_w_ (Scholander et al. 1964). The Ψ_w_ could be measured at different times of the day. If the measurement is taken at predawn, the soil and plant Ψ_w_ are in equilibrium, as plant transpiration is negligible (Jones 2006). When the midday measurement is performed on sun‐exposed leaves, the value obtained is the leaf Ψ_w_ (Ψ_w,leaf_). If the leaf is covered with an aluminium bag to prevent transpiration, the Ψ_w,leaf_ equilibrates with the stem Ψ_w_ (Ψ_w,stem_), so that the Ψ_w,stem_ is measured. Ψ_w,stem_ is considered the most used variable to determine plant water status for irrigation scheduling, as it is less sensitive to meteorological conditions (McCutchan and Shackel 1992; Naor 2000). For irrigation management, Ψ_w,stem_ thresholds have been established for several crops.

#### 6.4.1 Leaf Gas Exchange

Leaf gas exchange refers to the ratio of water vapour leaving the stomata, the *g*_s_, and the CO_2_ entering, thus the net photosynthesis (*A*_N_). It is now well known that stomatal closure is one of the first plant responses to drought, and it usually implies a reduction in the photosynthesis rate (Flexas and Medrano 2002).

Leaf gas exchange is a good indicator of a plant's water status in several crops, such as citrus (Pérez‐Pérez et al. 2009) or olive trees (Rodriguez‐Dominguez et al. 2019), and could be used for irrigation management.

#### 6.4.2 Canopy Temperature (*T*_c_)

Canopy temperature (*T*_c_) is a good indicator of water status, as it indirectly measures the degree of stomatal aperture. T_c_ is a non‐destructive parameter that can be measured in situ or remotely using infrared radiometer sensors or thermal cameras. Infrared thermography estimates the canopy temperature, which increases because of water stress. If plants suffer from water stress, stomata close, increasing the leaf temperature. In this way, thermography is a useful tool for irrigation scheduling in many crops (Berni et al. 2009). However, agrometeorological variations influence the thermal response of the canopy. For this reason, several thermal indices have been proposed to mitigate these effects. The crop water stress index (CWSI) is the most utilized. It is based on the relationship between T_c_, air temperature (*T*_a_), and VPD (Jackson et al. 1981). CWSI has been successfully used for monitoring the crop water status in citrus (Gonzalez‐Dugo et al. 2014), almond (García‐Tejero et al. 2012; García‐Tejero, Ortega‐Arévalo, et al. 2018; García‐Tejero, Rubio, et al. 2018; García‐Tejero, Gutiérrez‐Gordillo, et al. 2018), and olive trees (García‐Tejero et al. 2017).

#### 6.4.3 Trunk Diameter Variations

Linear trunk diameter variation is an indirect indicator of plant water status. Stem diameter experiments measure daily cycles of shrinkage and dilatation. Stem diameter is at its maximum before dawn and at its minimum in the afternoon. The difference in maximal stem diameter between consecutive days indicates trunk growth, while the magnitude of daily stem contraction or maximum daily shrinkage (MDS) is influenced by atmospheric evaporative demand and soil moisture levels. MDS can thus be a useful metric for irrigation scheduling. When compared to well‐watered reference trees in the same orchard, an increase in trunk MDS beyond certain threshold values can trigger the need for irrigation (Goldhamer and Fereres 2001). The use of MDS for irrigation management has been proven to be feasible (Mirás‐Avalos et al. 2017).

#### 6.4.4 Continuous Leaf Turgor Pressure

Leaf turgor is related to plant water status. The ZIM‐system (YARA ZIM Plant Technology, Hennigsdorf, Germany) is a magnetic‐based probe that measures the pressure (*P*_p_) transfer function through a patch of an intact leaf. This *P*_p_ has been shown to be inversely correlated with the Ψ_P_ (Zimmermann et al. 2008, 2010). The ZIM system provides a user‐friendly water stress index, suitable for deriving irrigation decisions solely based on visual analysis (Fernández 2014). This system has the potential for irrigation scheduling in olives (Ehrenberger et al. 2012; Fernández et al. 2011), and can be used to continuously monitor water status in Clementine and Persimmon trees under field conditions, improving irrigation timing and WUE (Ballester et al. 2017; Martínez‐Gimeno et al. 2017); lastly, it has been tested in additional species, such as banana trees to monitor daily turgor fluctuations (Zimmermann et al. 2010), in tobacco, to assess water stress responses revealing that plants subjected to Cl^−^ at macronutrient levels exhibited fewer drought‐related symptoms (Franco‐Navarro et al. 2021), and in wheat, to track real‐time turgor changes associated with transpiration and water uptake dynamics (Bramley et al. 2013).

### 6.5 Latest Advancements in Crop Monitoring for Precision Irrigation

Crop monitoring has become an essential component of precision irrigation, facilitating efficient water management in agriculture. Recent advancements have enabled accurate and continuous assessments of crop water status, ensuring optimal irrigation scheduling while preventing water stress and overwatering (Lakhiar et al. 2024). Among the latest innovations, microtensionmeters provide real‐time measurements of plant Ψ_w_ using microelectromechanical pressure sensors embedded in the trunk (Conesa et al. 2023). Non‐contact Resonant Ultrasonic Spectrometry (NC‐RUS) has also emerged as a non‐invasive technique to assess plant water status, offering early detection of water stress in crops such as citrus (Fariñas et al. 2021). Additionally, remote sensing technologies, including satellite imagery and aerial drones, allow for non‐invasive crop monitoring, optimizing irrigation by analyzing stress levels, water uptake, and photosynthetic activity (Martínez‐Peña et al. 2023). Despite their potential, challenges such as high costs and technical expertise requirements need to be addressed for widespread adoption. Microneedle sensors represent another frontier in precision irrigation, enabling real‐time ion concentration monitoring in plants with a high accuracy and a rapid response (Wang, Molinero‐Fernández, et al. 2024). These sensors are non‐destructive and durable, making them valuable tools for continuous ion transport tracking under water stress conditions. As technology and artificial intelligence advance, the integration of these innovative monitoring techniques will enhance precision irrigation (e.g., Ikos Raindrop Algorithm, https://help.ikosadvanced.com/doc/raindrop/), improving WUE and sustainability in agricultural practices. The following sections will explore these advancements in greater detail.

#### 6.5.1 Microtensionmeters

Microtensionmeters represent a viable option for the continuous monitoring of crop water status. These devices measure Ψ_w_ using a microelectromechanical pressure sensor capable of in situ measurements. Embedded in the trunk, microtensionmeters directly measure Ψ_w,stem_. This method is promising for determining Ψ_w,stem_, as it can be automated to provide continuous data in easy‐to‐interpret pressure units, similar to traditional pressure chamber methods (Blanco and Kalcsits 2021; Conesa et al. 2023).

#### 6.5.2 Non‐Contact Resonant Ultrasonic Spectrometry (NC‐RUS)

This innovative technique has emerged as a powerful tool to determine plant water status in a non‐destructive, non‐invasive, and rapid way (Gómez Álvarez‐Arenas et al. 2009). It has been found that ultrasonic parameters obtained immediately from field measurements correlate with Ψ_w_ and are also sensitive to the early detection of water stress in citrus crops, thus becoming a potential new water stress indicator for irrigation scheduling (Fariñas et al. 2021).

#### 6.5.3 Remote Sensing for Crop Monitoring

The development of new technologies based on remote sensing enables the quick and accurate acquisition of information about crops and the land surface. The use of remote sensing techniques for non‐invasive crop monitoring plays a crucial role in precision irrigation (Martínez‐Peña et al. 2023). Remote sensing provides spatial and spectral imagery data that can be used for crop monitoring and to optimize irrigation, resulting in water savings and improved crop physiology. It includes satellite imagery and aerial drones equipped with sensors. Satellite and drone‐based remote sensing platforms capture images, allowing for the monitoring of stress levels, water uptake patterns, chlorophyll content, photosynthetic activity, and even leaf area index (Huang et al. 2024; Zu et al. 2024). However, the implementation of remote sensing technologies comes with a few challenges. The high initial cost for acquiring and deploying these technologies can be a barrier, especially for small‐scale farmers. Additionally, the analysis and interpretation of data require technical expertise, which might not always be readily available. Weather conditions can also affect the accuracy of remote sensing data.

#### 6.5.4 Microneedle Sensors

A dual potentiometric K^+^/Na^+^ microneedle (MN) sensor has recently been developed for real‐time monitoring of ion concentrations in plants, demonstrating rapid and accurate responses under various stimuli (Wang, Molinero‐Fernández, et al. 2024). The MN patch demonstrated versatility across plant species and offered a high spatiotemporal resolution, representing a significant advancement for continuous ion monitoring in stressed plants. In addition, it also offers the following advantages: (i) non‐destructive monitoring; (ii) rapid response time; (iii) high accuracy and reliability; (iv) durability; (v) wide linear range; (vi) comparable with standard methods; and (vii) real‐time ion transport tracking.

### 6.6 Compilation of Key Points, Future Perspectives and Conclusion

Over the past few decades, significant progress has been made in understanding plant responses to drought stress, driven by advancements in physiology, molecular biology, genetics, and agronomic practices. Research has identified key physiological adaptations, such as stomatal regulation, osmotic adjustment, and antioxidant defenses, providing a foundation for genetic and biotechnological approaches to improve drought resilience. Breeding programs employing MAS, GWAS, and high‐throughput sequencing have successfully developed drought‐tolerant crop varieties with optimized root architectures, enhanced WUE, and improved metabolic responses under water‐limited conditions.

Biotechnological advancements, particularly the application of genome‐editing technologies such as CRISPR/Cas9, have enabled precise modifications of drought‐responsive genes, including *DREB*, *NAC*, and *WRKY* TFs, significantly enhancing plant stress tolerance. Epigenetic studies further suggest that stress memory mechanisms could be leveraged to develop crops with inheritable drought resilience. Moreover, synthetic biology approaches hold promise for designing genetic circuits that optimize water use and improve drought resilience.

On the agronomic front, sustainable water management strategies have emerged as essential tools for mitigating drought impacts. Precision irrigation techniques, including deficit irrigation and subsurface drip systems, have optimized water use while maintaining productivity. Soil conservation practices, such as mulching, cover cropping, and no‐till farming, have demonstrated their effectiveness in improving soil moisture retention and reducing water loss. Additionally, plant‐microbe interactions, particularly with beneficial rhizobacteria and mycorrhizal fungi, have shown potential in enhancing nutrient uptake and improving drought tolerance.

Integrated water management systems that combine irrigation scheduling, soil moisture conservation, and rainwater harvesting are gaining traction, optimizing resource use while minimizing waste. Advances in nano‐ and bio‐based water treatment technologies further contribute to water quality improvements, soil remediation, and nutrient delivery. Climate‐smart agricultural practices, such as agroforestry and sustainable land management, enhance resilience to climate variability by improving soil moisture retention and biodiversity. Remote sensing technologies, including satellite imagery and Unmanned Aerial Vehicles (UAVs), facilitate large‐scale assessments of crop health and water stress, enabling more efficient precision agriculture through machine learning and Geographic Information System (GIS) applications.

Future research directions should focus on integrating ‐omics technologies like genomics, proteomics, and metabolomics with artificial intelligence‐driven phenotyping platforms to accelerate the identification and breeding of drought‐adaptive traits. Systems biology approaches will be instrumental in modeling complex biological responses, while functional genomics studies can validate candidate genes for targeted crop improvement. Additionally, the exploration of beneficial microbial symbionts and their role in enhancing plant resilience through improved nutrient uptake and disease resistance warrants further investigation.

The development of multi‐stress tolerant crops is a critical area of research, acknowledging the interconnectedness of various environmental stressors. Trait stacking, involving the introgression of multiple stress tolerance traits, represents a promising strategy for improving overall plant resilience. Moreover, the use of machine learning and predictive modeling in breeding programs will enhance efficiency and adaptability to climate variability.

In conclusion, addressing the escalating challenges posed by drought stress in agriculture necessitates a multifaceted approach, integrating genetic, biotechnological, agronomic, and technological innovations. By leveraging precision irrigation, soil management strategies, breeding techniques, and cutting‐edge biotechnologies, researchers and agricultural practitioners can develop resilient cropping systems that ensure food security in the face of climate change. Interdisciplinary collaborations and continued research efforts will be vital in advancing sustainable agriculture, promoting WUE, and fostering global resilience to drought stress.

## Author Contributions

All authors conceived and performed the organization of the manuscript. J.D.F.‐N., Y.G.P., J.M.C.‐F., J.A.H., and J.R.A.‐M. participated in the writing and revisions of the entire article and specific chapters; Y.G.P., S.A., Á.C., J.M.C.‐F., M.J.G.‐B., J.A.H., I.M.‐A., C.P., J.G.P.‐P., M.J.S.‐B., and M.T. participated in the writing of specific chapters; J.D.F.‐N. composed all the figures; J.R.A.‐M. conceived the initial idea for writing the manuscript and organized the various working groups to write specific chapters.

## Conflicts of Interest

The authors declare no conflicts of interest.

## Supporting information


**Supplementary Figure S1.** Role of Cl^−^ in stomatal opening and closing.

**Supplementary Figure S2.** Schematic representation of Cl^−^ functions according to availability in the micro‐ or macronutrient range.

**Supplementary Table S1.** Sensitivity or tolerance of leaf tissues to Cl^−^ content.

## Data Availability

Data sharing is not applicable to this article as no new data were created or analyzed in this study. Our contribution is a review article.
