# Supplementary material for: Advancements in Water‐Saving Strategies and Crop Adaptation to Drought: A Comprehensive Review
Source: Physiol Plant. 2025 Jul 2;177(4):e70332. doi: 10.1111/ppl.70332 (PMC12215295; doi:10.1111/ppl.70332)
Supplement: Supplementary file 1 — Supplementary Figure S1. Role of Cl− in stomatal opening and closing. [file PPL-177-e70332-s002.docx]

**Supplementary figure S1. Role of Cl^-^ in stomatal opening and closing.**

**Supplementary figure S1. Role of Cl^-^ in stomatal opening and closing.** Representation of the leaf surface, and the open and closed state of the stomatal pore under light and dark conditions, respectively. Scanning electron microscopy images was carried out by Dr. M. Rosario Álvarez Morales. Optical microscopy images of stomata (60X), performed on cultured leaf peelings, were published in Franco-Navarro et al. (2019). (B) Scheme that describes the opening and closing mechanism of the stomata. The pumping of protons (H^+^) is carried out in response to the presence of light, specifically blue light, and entails expenditure of energy (ATP). This hyperpolarizes the PM, promoting the transport of potassium (K^+^) into the cell, and in turn the co-transport with H^+^ of chloride (Cl^-^) and/or nitrate (NO_3_^-^) through the proton-motive force (PMF). On the other hand, the release of malate (Mal^2-^) from starch reserves is activated. Both Mal^2-^ and NO_3_^-^, and especially Cl^-^, are transported to the vacuole, promoting the accumulation of water (H_2_O), producing the conformational change that opens the pore. Stomatal closure occurs in the absence of light and/or the presence of abscisic acid (ABA) through the opening of anion channels, producing a depolarization of the MP, which in turn induces the opening of K^+^ channels, producing a massive outflow of ions, and therefore, water. A reconversion of Mal^2-^ into starch (gluconeogenesis) is also induced (Hedrich and Geiger, 2017; Konrad et al., 2018; Roux and Leonhardt, 2018; Saito and Uozumi, 2019). (C) Scheme that describes the stomatal closure in guard cells driven by ABA signalling pathway triggered by drought conditions. ABA-induced responses include Ca^2+^ influx, ROS production, and ion channel regulation, leading to water conservation through stomatal closure to prevent water loss. The diagram illustrates key channels and transporters, including: (i) ABA Importer that facilitates ABA uptake, activating downstream signalling cascades. (ii) Ca^2+^ channels control Ca^2+^ influx, a central signal in ABA responses. (iii) SLAC1 (S-type anion channel) facilitates Cl^-^ and NO_3_^-^ efflux, leading to membrane depolarization. (iv) ALMT12/QUAC1 (S-type anion channel) contributes to anion efflux and stomatal closure, and (v) GORK (K^+^ outward rectifying channel) mediates K^+^ efflux, reducing turgor pressure in guard cells. For a more comprehensive and deeper understanding of the whole set of channels and transporters in guard cells see for instance the work of Liu et al. (2022) and Zuo et al. (2025). Abbreviations: Chloroplasts, CH; epidermal cell, EC; guard cells, CG; nucleus, N; vacuole, V. Parts of the images were fully provided with permission from J.D. Franco-Navarro's thesis (Franco-Navarro, 2022). Most elements of this scheme were created with BioRender.com (CC-BY 4.0 license).

This Supplementary Figure is included in the paper titled: ‘***Advancements in water-saving strategies and crop adaptation to drought: A comprehensive review*’** (*Physiologia Plantarum*) by Juan D. Franco-Navarro, Yaiza Gara Padilla, Sara Álvarez, Ángeles Calatayud, José Manuel Colmenero-Flores, María José Gómez-Bellot, José Antonio Hernández, Isabel Martínez-Alcalá, Consuelo Penella, Juan Gabriel Pérez-Pérez, María Jesús Sánchez-Blanco, María Tasa, and José Ramón Acosta-Motos.

**References:**

Franco-Navarro, J.D., 2022. Funciones del cloruro como macronutriente beneficioso en plantas superiores. University of Seville. https://doi.org/10.13140/RG.2.2.13246.15680/1

Franco-Navarro, J.D., Rosales, M.A., Cubero-Font, P., Calvo, P., Álvarez, R., Diaz-Espejo, A., Colmenero-Flores, J.M., 2019. Chloride as a macronutrient increases water-use efficiency by anatomically driven reduced stomatal conductance and increased mesophyll diffusion to CO2. Plant J. 99, 815–831. https://doi.org/10.1111/tpj.14423

Hedrich, R., Geiger, D., 2017. Biology of SLAC1-type anion channels - from nutrient uptake to stomatal closure. New Phytol. 216, 46–61. https://doi.org/10.1111/nph.14685

Konrad, K.R., Maierhofer, T., Hedrich, R., 2018. Spatio-temporal aspects of Ca2+ signalling: lessons from guard cells and pollen tubes. J. Exp. Bot. 69, 4195–4214. https://doi.org/10.1093/jxb/ery154

Liu, H., Song, S., Zhang, H., Li, Y., Niu, L., Zhang, J., Wang, W., 2022. Signaling Transduction of ABA, ROS, and Ca2+ in Plant Stomatal Closure in Response to Drought. Int. J. Mol. Sci. 23, 14824. https://doi.org/10.3390/ijms232314824

Roux, B., Leonhardt, N., 2018. The Regulation of Ion Channels and Transporters in the Guard Cell, in: Maurel, C. (Ed.), Membrane Transport in Plants. Academic Press Ltd-Elsevier Science Ltd, London, pp. 171–214. https://doi.org/10.1016/bs.abr.2018.09.013

Saito, S., Uozumi, N., 2019. Guard Cell Membrane Anion Transport Systems and Their Regulatory Components: An Elaborate Mechanism Controlling Stress-Induced Stomatal Closure. Plants (Basel, Switzerland) 8, 9. https://doi.org/10.3390/plants8010009

Zuo, Y., Abbas, A., Dauda, S.O., Chen, C., Bose, J., Donovan-Mak, M., Wang, Y., He, J., Zhang, P., Yan, Z., Chen, Z.-H., 2025. Function of key ion channels in abiotic stresses and stomatal dynamics. Plant Physiol. Biochem. 220, 109574. https://doi.org/10.1016/j.plaphy.2025.109574
