# Supplementary material for: Advancements in Water‐Saving Strategies and Crop Adaptation to Drought: A Comprehensive Review
Source: Physiol Plant. 2025 Jul 2;177(4):e70332. doi: 10.1111/ppl.70332 (PMC12215295; doi:10.1111/ppl.70332)
Supplement: Supplementary file 2 — Supplementary Figure S2. Schematic representation of Cl− functions according to availability in the micro‐ or macronutrient range. [file PPL-177-e70332-s003.docx]

**Supplementary figure S2. Schematic representation of Cl^-^ functions according to availability in the micro- or macronutrient range.**

Different biological functions are defined according to the external Cl^-^ application (0-5 mM Cl^-^) or the resulting leaf bulk tissue concentration (1-110 mM Cl^-^). Functions indicated in the 0.0-0.1 mM Cl^-^ treatment range summarises previous knowledge of Cl^-^ roles as an essential micronutrient (Broyer et al., 1954; Johnson et al., 1957; White and Broadley, 2001; Xu et al., 2000); biological functions indicated in the 0.1-5.0 mM Cl^-^ treatment range summarise the recent knowledge on the specific functions of Cl^-^ as a beneficial macronutrient in higher plants (Bazihizina et al., 2019; Cakmak et al., 2022; Colmenero-Flores et al., 2019; Cram, 1983; Cui et al., 2020; Franco-Navarro, 2022; Franco-Navarro et al., 2021, 2019, 2016; Geilfus, 2018a, 2018b, 2019; Lucas et al., 2024; Maron, 2019; Peinado-Torrubia et al., 2023; Raven, 2017; Rosales, et al., 2020a; Rosales et al., 2020b). Abbreviations: Chloride, Cl^-^; chloroplast surface area exposed to intercellular air spaces, *S*_c_; mesophyll conductance to CO_2_ diffusion, *g*_m_; mesophyll surface area exposed to intercellular air spaces, *S*_mes_; nitrate, NO_3_^-^; nitrogen uptake efficiency, NU_P_E; nitrogen utilization efficiency, NU_T_E; oxygen evolution complex of the photosystem II, OEC-PSII; stomatal conductance, *g*_s_; stomatal index, SI; water use efficiency, WUE. The schematic representation was fully provided with permission from J.D. Franco-Navarro's thesis (Franco-Navarro, 2022).

This Supplementary Figure is included in the paper titled: ‘***Advancements in water-saving strategies and crop adaptation to drought: A comprehensive review*’** (*Physiologia Plantarum*) by Juan D. Franco-Navarro, Yaiza Gara Padilla, Sara Álvarez, Ángeles Calatayud, José Manuel Colmenero-Flores, María José Gómez-Bellot, José Antonio Hernández, Isabel Martínez-Alcalá, Consuelo Penella, Juan Gabriel Pérez-Pérez, María Jesús Sánchez-Blanco, María Tasa, and José Ramón Acosta-Motos.

**References:**

Bazihizina, N., Colmer, T.D., Cuin, T.A., Mancuso, S., Shabala, S., 2019. Friend or Foe? Chloride Patterning in Halophytes. Trends Plant Sci. 24, 142–151. https://doi.org/10.1016/j.tplants.2018.11.003

Broyer, T.C., Carlton, A.B., Johnson, C.M., Stout, P.R., 1954. Chlorine-A micronutrient element for higher plants. Plant Physiol. 29, 526–532.

Cakmak, I., Brown, P., Colmenero-Flores, J.M., Husted, S., Kutman, B., Nikolic, M., Rengel, Z., Schmidt, S.B., Zhao, F., 2022. Chapter 7 - Function of Nutrients: Micronutrients, in: Marschner’s Mineral Nutrition of Higher Plants (Fourth Edition). Academic Press Ltd-Elsevier Science Ltd, p. 725.

Colmenero-Flores, J.M., Franco-Navarro, J.D., Cubero-Font, P., Peinado-Torrubia, P., Rosales, M.A., 2019. Chloride as a Beneficial Macronutrient in Higher Plants: New Roles and Regulation. Int. J. Mol. Sci. 20. https://doi.org/10.3390/ijms20194686

Cram, W.J., 1983. Chloride Accumulation as a Homeostatic System: Set Points and Perturbations: the physiological significance of influx isotherms, temperature effects and the influence of plant growth substances. J. Exp. Bot. 34, 1484–1502. https://doi.org/10.1093/jxb/34.11.1484

Cui, Y.-N., Li, X.-T., Yuan, J.-Z., Wang, F.-Z., Guo, H., Xia, Z.-R., Wang, S.-M., Ma, Q., 2020. Chloride is beneficial for growth of the xerophyte Pugionium cornutum by enhancing osmotic adjustment capacity under salt and drought stresses. J. Exp. Bot. 71, 4215–4231. https://doi.org/10.1093/jxb/eraa158

Franco-Navarro, J.D., 2022. Funciones del cloruro como macronutriente beneficioso en plantas superiores. University of Seville. https://doi.org/10.13140/RG.2.2.13246.15680/1

Franco-Navarro, J.D., Brumós, J., Rosales, M.A., Cubero-Font, P., Talón, M., Colmenero-Flores, J.M., 2016. Chloride regulates leaf cell size and water relations in tobacco plants. J. Exp. Bot. 67, 873–891. https://doi.org/10.1093/jxb/erv502

Franco-Navarro, J.D., Díaz-Rueda, P., Rivero-Núñez, C.M., Brumós, J., Rubio-Casal, A.E., de Cires, A., Colmenero-Flores, J.M., Rosales, M.A., 2021. Chloride nutrition improves drought resistance by enhancing water deficit avoidance and tolerance mechanisms. J. Exp. Bot. 72, 5246–5261. https://doi.org/10.1093/jxb/erab143

Franco-Navarro, J.D., Rosales, M.A., Cubero-Font, P., Calvo, P., Álvarez, R., Diaz-Espejo, A., Colmenero-Flores, J.M., 2019. Chloride as a macronutrient increases water-use efficiency by anatomically driven reduced stomatal conductance and increased mesophyll diffusion to CO2. Plant J. 99, 815–831. https://doi.org/10.1111/tpj.14423

Geilfus, C.-M., 2019. Chloride in soil: From nutrient to soil pollutant. Environ. Exp. Bot. 157, 299–309. https://doi.org/10.1016/j.envexpbot.2018.10.035

Geilfus, C.-M., 2018a. Chloride: from Nutrient to Toxicant. Plant Cell Physiol. 59, 877–886. https://doi.org/10.1093/pcp/pcy071

Geilfus, C.-M., 2018b. Review on the significance of chlorine for crop yield and quality. Plant Sci. 270, 114–122. https://doi.org/10.1016/j.plantsci.2018.02.014

Johnson, C.M., Stout, P.R., Broyer, T.C., Carlton, A.B., 1957. Comparative chlorine requirements of different plant species. Plant Soil 8, 337–353.

Lucas, M., Diaz-Espejo, A., Romero-Jimenez, D., Peinado-Torrubia, P., Delgado-Vaquero, A., Álvarez, R., Colmenero-Flores, J.M., Rosales, M.A., 2024. Chloride reduces plant nitrate requirement and alleviates low nitrogen stress symptoms. Plant Physiol. Biochem. 212, 108717. https://doi.org/10.1016/j.plaphy.2024.108717

Maron, L.G., 2019. From foe to friend: the role of chloride as a beneficial macronutrient. Plant J. 99, 813–814. https://doi.org/10.1111/tpj.14498

Peinado-Torrubia, P., Álvarez, R., Lucas, M., Franco-Navarro, J.D., Durán-Gutiérrez, F.J., Colmenero-Flores, J.M., Rosales, M.A., 2023. Nitrogen assimilation and photorespiration become more efficient under chloride nutrition as a beneficial macronutrient. Front. Plant Sci. 13. https://doi.org/10.3389/fpls.2022.1058774

Raven, J.A., 2017. Chloride: essential micronutrient and multifunctional beneficial ion. J. Exp. Bot. 68, 359–367. https://doi.org/10.1093/jxb/erw421

Rosales, M.A., Franco-Navarro, J.D., Moreno Racero, F.J., Colmenero-Flores, J.M., 2020. Beneficios de una fertilización rica en cloruro para la agricultura y sus efectos en la salud humana. Hortic. 4 42–47.

Rosales, M.A., Franco-Navarro, J.D., Peinado-Torrubia, P., Díaz-Rueda, P., Álvarez, R., Colmenero-Flores, J.M., 2020. Chloride Improves Nitrate Utilization and NUE in Plants. Front. Plant Sci. 11, 154–166. https://doi.org/10.3389/fpls.2020.00442

White, P.J., Broadley, M.R., 2001. Chloride in soils and its uptake and movement within the plant: A review. Ann. Bot. 88, 967–988. https://doi.org/10.1006/anbo.2001.1540

Xu, G.H., Magen, H., Tarchitzky, J., Kafkafi, U., 2000. Advances in chloride nutrition of plants, in: Sparks, D.L. (Ed.), Advances in Agronomy, Vol 68. pp. 97–150.
