# Supplementary material for: Advancements in Water‐Saving Strategies and Crop Adaptation to Drought: A Comprehensive Review
Source: Physiol Plant. 2025 Jul 2;177(4):e70332. doi: 10.1111/ppl.70332 (PMC12215295; doi:10.1111/ppl.70332)
Supplement: Supplementary file 3 — Supplementary Table S1. Sensitivity or tolerance of leaf tissues to Cl− content. [file PPL-177-e70332-s001.docx]

**Supplementary Table S1. Sensitivity or tolerance of leaf tissues to Cl^-^ content.**

| **Supplementary table S1**. **Sensitivity or tolerance of leaf tissues to Cl^-^ content.** | | |
| --- | --- | --- |
| **Species** | **Condition** | **References** |
| **Cotton** | Sensitive | (Fan et al., 2016) |
| **Fruit trees** |  | (Bar et al., 1997; Bell et al., 1997; Robinson, 1986) |
| **Peanuts** |  | (Wang et al., 1989) |
| **Strawberries** |  | (Robinson, 1986; Wang et al., 1989) |
| **Barley** | Tolerant | (Engel et al., 1997, 1994) |
| **Lettuce** |  | (Johnson et al., 1957; Wei et al., 1989) |
| **Potato** |  | (Bernstein et al., 1951; Corbett and Gausman, 1960; James et al., 1970) |
| **Spinach** |  | (Robinson and Downton, 1984) |
| **Sugar beet** |  | (Terry, 1977; Ulrich and Ohki, 1956; Zhou and Zhang, 1992) |
| **Tobacco** |  | (Colmenero-Flores et al., 2019; Franco-Navarro et al., 2021, 2019, 2016; Rosales et al., 2020a; Rosales et al., 2020b) |
| **Tomato** |  | (Broyer et al., 1954; Kafkafi et al., 1982) |
| It has been reported that leaf Cl^-^ contents greater than 3.5 mg Cl^-^ g^-1^ DW of the leaf, is toxic to sensitive species (*i.e.*, some fruit tress; Bar et al., 1997; Bell et al., 1997; Robinson, 1986). On the contrary, 20-50 mg Cl^-^ g^-1^ DW leaf are not harmful to tolerant species (*i.e.*, tomato; Broyer et al., 1954; Kafkafi et al., 1982). | | |

This Supplementary Table is included in the paper titled: ‘***Advancements in water-saving strategies and crop adaptation to drought: A comprehensive review*’** (*Physiologia Plantarum*) by Juan D. Franco-Navarro, Yaiza Gara Padilla, Sara Álvarez, Ángeles Calatayud, José Manuel Colmenero-Flores, María José Gómez-Bellot, José Antonio Hernández, Isabel Martínez-Alcalá, Consuelo Penella, Juan Gabriel Pérez-Pérez, María Jesús Sánchez-Blanco, María Tasa, and José Ramón Acosta-Motos.

**References:**

Bar, Y., Apelbaum, A., Kafkafi, U., Goren, R., 1997. Relationship between chloride and nitrate and its effect on growth and mineral composition of avocado and citrus plants. J. Plant Nutr. 20, 715–731. https://doi.org/10.1080/01904169709365288

Bell, P.F., Vaughn, J.A., Bourgeois, W.J., 1997. Leaf analysis finds high levels of chloride and low levels of zinc and manganese in Louisiana citrus. J. Plant Nutr. 20, 733–743. https://doi.org/10.1080/01904169709365289

Bernstein, L., Ayers, A.D., Wadleigh, C.H., 1951. The salt tolerance of white rose potatoes, in: Proceedings of the American Society for Horticultural Science. pp. 231–236.

Broyer, T.C., Carlton, A.B., Johnson, C.M., Stout, P.R., 1954. Chlorine-A micronutrient element for higher plants. Plant Physiol. 29, 526–532.

Colmenero-Flores, J.M., Franco-Navarro, J.D., Cubero-Font, P., Peinado-Torrubia, P., Rosales, M.A., 2019. Chloride as a Beneficial Macronutrient in Higher Plants: New Roles and Regulation. Int. J. Mol. Sci. 20. https://doi.org/10.3390/ijms20194686

Corbett, E.G., Gausman, H.W., 1960. The Interaction of Chloride with Sulfate and Phosphate in the Nutrition of Potato Plants (Solanum tuberosum). Agron. J. 52, 94–96. https://doi.org/10.2134/agronj1960.00021962005200020011x

Engel, R.E., Bruckner, P.L., Mathre, D.E., Brumfield, S.K.Z., 1997. A Chloride-Deficient Leaf Spot Syndrome of Wheat. Soil Sci. Soc. Am. J. 61, 176–184. https://doi.org/10.2136/sssaj1997.03615995006100010026x

Engel, R.E., Eckhoff, J., Berg, R.K., 1994. Grain Yield, Kernel Weight, and Disease Responses of Winter Wheat Cultivars to Chloride Fertilization. Agron. J. 86, 891–896. https://doi.org/10.2134/agronj1994.00021962008600050025x

Fan, X., Tang, Z., Tan, Y., Zhang, Y., Luo, B., Yang, M., Lian, X., Shen, Q., Miller, A.J., Xu, G., 2016. Overexpression of a pH-sensitive nitrate transporter in rice increases crop yields. Proc. Natl. Acad. Sci. 113, 7118–7123. https://doi.org/10.1073/pnas.1525184113

Franco-Navarro, J.D., Brumós, J., Rosales, M.A., Cubero-Font, P., Talón, M., Colmenero-Flores, J.M., 2016. Chloride regulates leaf cell size and water relations in tobacco plants. J. Exp. Bot. 67, 873–891. https://doi.org/10.1093/jxb/erv502

Franco-Navarro, J.D., Díaz-Rueda, P., Rivero-Núñez, C.M., Brumós, J., Rubio-Casal, A.E., de Cires, A., Colmenero-Flores, J.M., Rosales, M.A., 2021. Chloride nutrition improves drought resistance by enhancing water deficit avoidance and tolerance mechanisms. J. Exp. Bot. 72, 5246–5261. https://doi.org/10.1093/jxb/erab143

Franco-Navarro, J.D., Rosales, M.A., Cubero-Font, P., Calvo, P., Álvarez, R., Diaz-Espejo, A., Colmenero-Flores, J.M., 2019. Chloride as a macronutrient increases water-use efficiency by anatomically driven reduced stomatal conductance and increased mesophyll diffusion to CO2. Plant J. 99, 815–831. https://doi.org/10.1111/tpj.14423

James, D.W., Weaver, W.H., Reeder, R.L., 1970. Chloride uptake by potatoes and the effects of potassium chloride, nitrogen and phosphorus fertilization. Soil Sci. 109.

Johnson, C.M., Stout, P.R., Broyer, T.C., Carlton, A.B., 1957. Comparative chlorine requirements of different plant species. Plant Soil 8, 337–353.

Kafkafi, U., Valoras, N., Letey, J., 1982. Chloride interaction with nitrate and phosphate nutrition in tomato ( Lycopersicon esculentum L.). J. Plant Nutr. 5, 1369–1385. https://doi.org/10.1080/01904168209363070

Robinson, J.B., 1986. Fruits, vines and nuts. In’Plant Analysis-an Interpretation Manual’.(Eds D. J. Reuter and J. B. Robinson.) pp. 120-47.

Robinson, S.P., Downton, W.J.S., 1984. Potassium, sodium, and chloride content of isolated intact chloroplasts in relation to ionic compartmentation in leaves. Arch. Biochem. Biophys. 228, 197–206. https://doi.org/10.1016/0003-9861(84)90061-4

Rosales, M.A., Franco-Navarro, J.D., Moreno Racero, F.J., Colmenero-Flores, J.M., 2020. Beneficios de una fertilización rica en cloruro para la agricultura y sus efectos en la salud humana. Hortic. 4 42–47.

Rosales, M.A., Franco-Navarro, J.D., Peinado-Torrubia, P., Díaz-Rueda, P., Álvarez, R., Colmenero-Flores, J.M., 2020. Chloride Improves Nitrate Utilization and NUE in Plants. Front. Plant Sci. 11, 154–166. https://doi.org/10.3389/fpls.2020.00442

Terry, N., 1977. Photosynthesis, growth, and role of chloride. Plant Physiol. 60, 69–75. https://doi.org/10.1104/pp.60.1.69

Ulrich, A., Ohki, K., 1956. Chlorine, Bromine and Sodium As Nutrients for Sugar Beet Plants. Plant Physiol. 31, 171–181. https://doi.org/10.1104/pp.31.3.171

Wang, D.Q., Guo, B.C., Dong, X.Y., 1989. Toxicity effects of chloride on crops. Chin. J. Soil Sci. 30, 258–261.

Wei, S.Q., Zhou, Z.F., Liu, C., 1989. Effects of chloride on yield and quality of lettuce and its critical value of tolerance. Chin. J. Soil Sci 30, 262–264.

Zhou, B.K., Zhang, X.Y., 1992. Effects of chloride on growth and development of sugarbeet. Soil Fert 3, 41–43.
